# Using selfies to challenge public stereotypes of scientists

**DOI:** 10.1371/journal.pone.0216625

**Published:** 2019-05-10

**Authors:** Paige Brown Jarreau, Imogene A. Cancellare, Becky J. Carmichael, Lance Porter, Daniel Toker, Samantha Z. Yammine

**Affiliations:** 1 LifeOmic, Indianapolis, Indiana, United States of America; 2 College of Science, Communication across the Curriculum, LSU, Baton Rouge, Louisiana, United States of America; 3 Department of Entomology and Wildlife Ecology, University of Delaware, Newark, Delaware, United States of America; 4 Manship School of Mass Communication, LSU, Baton Rouge, Louisiana, United States of America; 5 Helen Wills Neuroscience Institute, UC Berkeley, Berkeley, California, United States of America; 6 Donnelly Centre for Cellular and Biomolecular Research, University of Toronto, Toronto, Ontario, Canada; USC Keck School of Medicine, Institute for Global Health, UNITED STATES

## Abstract

In an online Qualtrics panel survey experiment (N = 1620), we found that scientists posting self-portraits (“selfies”) to Instagram from the science lab/field were perceived as significantly warmer and more trustworthy, and no less competent, than scientists posting photos of only their work. Participants who viewed scientist selfies, especially posts containing the face of a female scientist, perceived scientists as significantly warmer than did participants who saw science-only images or control images. Participants who viewed selfies also perceived less symbolic threat from scientists. Most encouragingly, participants viewing selfies, either of male or female scientists, did not perceive scientists as any less competent than did participants viewing science-only or control images. Subjects who viewed female scientist selfies also perceived science as less exclusively male. Our findings suggest that self-portraiture by STEM professionals on social media can mitigate negative attitudes toward scientists.

## Introduction

There is growing awareness within the scientific community that researchers and their institutions can and should make public engagement and communication more of a priority [1–3]. But while scientists are answering calls to leverage new media tools [4], it is important for them to consider that not all science communication efforts are created equal. The impacts of these efforts depend on a variety of factors including real or perceived characteristics of the message (content, tone, frame, etc.), the audience(s), and the communicator(s) [5]. People tend to be cognitive misers in evaluating information and forming attitudes [6]. They often use a communicator’s perceived trustworthiness as a heuristic for how credible, personally relevant, reliable, pertinent, and persuasive a message is [7, 8].

People are more likely to pay attention to and act upon “quality information communicated by sources they see as trustworthy in terms of expertise, honesty, and shared identity” [5] and from sources they “like” [9]. Trust, which involves mutual understanding between communicator and audience, is vital for scientists speaking on issues of pressing public concern such as public health or climate change [10, 11]. The audience member should feel that scientists “get me”. Scientists and their institutions need to be sincerely friendly, demonstrate a shared value system, and engage people’s emotions [12]. High quality interactions with likeable scientists encourage positive beliefs about science and scientists’ messages [3].

Unfortunately, scientists are generally perceived by Americans as highly competent but only moderately warm; they “earn respect but not trust” [12]. Studies investigating scientists’ prioritization of public communication objectives have also found very little attention to this issue of warmth or focus on building trust [3, 13]. Scientists’ isolation in “the ivory tower” [2, 14] and focus on defending science rather than building relationships with the public [3], along with stereotype-reinforcing media portrayals, have likely contributed to both ambivalent public perceptions and more negative stereotypes of scientists.

Scientists’ use of direct-to-public communication tools such as social media [15] may be spurring concern over their public image. However, social media could also be an avenue for relationship building between scientists and citizens. These relationships could help challenge stereotypes of scientists as being competent but socially inept and eccentric [16], cold, and capable of immoral conduct [17]. To accomplish this, scientists must use social media in ways that communicate their warmth and not just their scientific competence.

## Background and literature review

### Social cognition, warmth and competence

We use social cognition and the **stereotype content model** [18] as the framework to understand how perceptions of scientist Instagrammers may impact trust in scientists. Social cognition consists of the idea that “people everywhere differentiate each other by liking (warmth, trustworthiness) and by respecting (competence, efficiency)” [19]. The stereotype content model postulates that group stereotypes can be captured by the two universal dimensions of social cognition: warmth and competence [18]. Warmth is based on one’s perceptions of another individual’s or group’s intentions, while competence is based on one’s confidence that the individual or group can act on their intentions. Traits that add to perceived warmth include friendliness, tolerance, modesty, openness, sociability, honesty, happiness, helpfulness, sincerity, and morality. Traits that add to perceived competence include intelligence, skill, creativity, and persistence [19]. Both competence and warmth are critical constructs of trust. However, perceived warmth carries more weight in terms of people’s attitudes and behaviors toward an individual or stereotyped group [19]. Morality or warmth judgments are primary—they are made more quickly, they determine approach or avoidance behavior, and they carry more weight in attitude formation than competence judgments do [19–21].

Outgroups (people unlike oneself) are often perceived as either competent but not warm and associated with feelings of threat and envy, neither competent nor warm and associated with feelings of contempt, or warm but not competent and thus pitied [19]. How one perceives outgroups can be predicted by the perceived status of the group and competition with one’s in-group. High status and competitive outgroups such as scientists are often perceived as competent but not very warm [12]. In-group individuals, close allies, and other groups like nurses and teachers are admired and perceived as both highly warm and competent [12]. Instead of threatening, they are “like you” in their underlying interests and values [19, 22, 23]. They have high potential to be compelling communicators. By sharing their values and humanizing themselves, we think scientists can get there too.

### Perceptions of scientists

Longitudinal results from the General Social Survey (GSS) since the 1970s suggest relatively stable and high levels of U.S. public confidence in scientists. Men, younger individuals, and individuals with higher levels of education and income express the highest levels of confidence in scientists. However, an increasing percentage of the U.S. public also finds scientists to be “odd and peculiar” (52% strongly agreed or agreed, up from 36% in 2012) [24]. This stereotype reflects longtime fiction media portrayals of scientists as eccentric, strange and detached from everyday life [25, 26]. Based on this and other stereotypes, scientists as a group don’t immediately inspire friendliness and warmth, vital characteristics of trusted communicators.

We should note that media portrayals of scientists have improved in the last decade toward characters that can generally be categorized as intelligent and “good” [27]. However, portrayals of scientists in both entertainment and news media still lack diversity, particularly in terms of gender and race according to the recent “Portray Her” report from The Lyda Hill Foundation and the Geena Davis’s Institute on Gender in Media. Popular images of the scientist as an elderly, white-haired man and people’s general unfamiliarity with real-life scientists and what they do tend to maintain negative stereotypes or at least perceptions that scientists are among an elite outgroup that doesn’t share the values, interests, and identity of most people.

### Gender science stereotypes, female scientists in the media, and warmth stereotypes

Warmth and competence stereotypes of scientists are likely exacerbated by gender stereotypes and gender science stereotypes that associate STEM with being male [28]. Women are generally perceived as more warm, likeable, and friendly, but slightly less competent, than men [7, 18]. Positive perceptions of female scientists as not only competent but also warm could be a positive force for scientist stereotypes, except that gender science stereotypes limit opportunities for women to succeed, become leaders in their fields, and positively influence the image of science. Pervasive stereotypes associate science and scientific work with men more than women [29]. These stereotypes both aggravate and are aggravated by gender gaps in retention, achievement, visibility, and leadership in STEM [30–37]. The Catch-22 for scientists’ public image problem is that women scientists who could improve perceptions of scientists’ relatability and warmth are both underrepresented in prestigious scientific media outlets [38] and in media that nonscientists consume [39]. They are also judged for taking on stereotypically male roles and traits associated with scientific work [35, 40, 41]. The clashing of gender roles in STEM is exemplified by the fact that female scientists are often told that they “don’t look like scientists” [42]. Independent, self-confident, competitive, senior women in male-dominated fields are harshly critiqued [43]. This makes it difficult for women to achieve the equal representation in STEM and the media attention that could help shift stereotypes of scientists and help young girls see themselves in STEM. Scientists from other marginalized groups face similar challenges.

The scientist as we’ve known “him” from literature is a white male, a social outsider who is unscrupulous or even dangerous in his pursuit of scientific discovery [44]. More modern media portrayals of scientists also tend to be male. Female scientists are often presented in media programs as more of an exception to the rule (a lonely heroine, an unlikely clever beauty, a rare superstar) than as positive counter-stereotypes [45]. Media portrayals of female scientists are likely to show them in supporting roles to male scientists and to focus on their appearance, sexuality, and personal lives as opposed to their work successes [46]. A fetishizing of the appearance of female scientists in the media reinforces gender role beliefs and promotes the idea that women in STEM are eye candy rather than experts. This was exemplified by the #distractinglysexy social media campaign that became viral in 2015 [47]. Media misrepresentation undermines women scientists’ ability to positively influence the stereotypical image of scientists as aloof, nerdy, socially inept, and male.

Social media platforms may offer women and minority scientists an opportunity to create their own narratives through self-images and new takes on what scientists look like, what they do, what they care about, and how both warm *and* competent they are. A diversity of real-life scientist faces in our media environments could help others begin to individuate scientists, help break down stereotypes, and foster more inclusion in STEM.

### Using visuals to put a friendly face on science and change stereotypes

Visual social networking platforms cater to real-time self-disclosure, which is critical to the relationship building that can promote trust between individuals and break down group stereotypes [48, 49]. One way to operationalize how a scientist could visually communicate their warmth is through today’s cultural artifact for online self-disclosure—the “selfie”. Selfies close the distance between photographer and viewer—even astronauts take selfies [50]. There is an emerging trend within the online STEM community to use portraits to help change people’s perceptions of what a scientist looks like and does on a daily basis. Hashtag movements such as #ThisIsWhatAScientistLooksLike, #BlackandSTEM, #DistractinglySexy, and #ScientistsWhoSelfie have attempted to combat stereotypes of STEM professionals through visual imagery [51]. While these movements may promote feelings of empowerment within the group seeking to change its public image, it is unknown whether such movements can actually improve stereotyped perceptions of scientists.

The idea of using portraits to change perceptions has a basis in social cognition research. Counterstereotypical exemplars have been shown to change perceptions of and bias toward outgroups, as well as reduce or revise stereotypes [52]. Individuation processes, in which viewers pay attention to a person’s individual attributes and see that person as human with ideally shared beliefs and good intentions [53], can help counter negative stereotypes about that person and their group. But individuating takes effort [54]. People often don’t put this effort toward stereotyped outgroups or groups they aren’t familiar with. However, individuation can take place when people are asked or motivated to take notice of individual attributes or pay extra attention to individual faces [53]. Individual processing of scientists’ faces, especially when they display warmth attributes, might help challenge stereotypes.

Positive facial expressions [55], images of friendly faces [56], and images of attractive faces [57] can influence social cognition. Smiling faces vs. neutral faces score higher on attributes including friendliness [58], generosity, agreeableness, sincerity [59], extroversion [55], and warmth [60]. Smiling can also combine with gender to influence perceptions of warmth. In one study, female smiling faces were evaluated as warmer than male smiling faces [56]. But while facial expressions can influence interpersonal trait inferences, it is unclear whether they can, when displayed by members of outgroups, move the needle on warmth stereotypes. There is evidence that positive images of individuals can suppress automatic attitudes and stereotypes [61], that social exposure to counterstereotypes (like women leaders) can reduce automatic gender stereotyping [62], and that counterstereotypic mental imagery (e.g. imagining a strong woman) can reduce implicit stereotypes [63].

In the study investigating exposure to counterstereotypic women leaders [62], exposure to a greater number of women faculty and deans over time had long-term impacts (on the range of at least a year, with ongoing exposure) on automatic gender stereotypes. Short-term exposures to counterstereotypic scientists via media stories or social media are likely to have less long-term impacts on scientist and science gender stereotypes. However, these exposures might have greater impact if viewers discovered and then followed counterstereotypic (e.g. female) scientists on Instagram, for example, thus seeing more from these scientists over time and even developing relationships with them. The relative impacts of repeated exposure to counterstereotypic scientists on Instagram as opposed to one-time exposure are out of the scope of this study, but should be investigated in future research, especially if a one-time exposure can produce significant changes in social cognitions.

### Why study Instagram as a tool for building trust in science

Instagram (IG), released in 2010, is a photo and video social media sharing platform that encourages viewers to enter Instagrammers’ worlds and get to know them through selfies and self-videos. Scientists, including several authors of this paper, are using IG to open up their lab and fieldwork for broader audiences. An interconnected group of thousands of scientists at various career stages use IG to share their lives and work as scientists, for example via #weareSTEMsquad. A number of popular “Rotation Curation” accounts feature different scientists through images and captions about their work, career paths, hobbies, etc. Through the use of hashtags, users can identify and follow trending topics.

Scientists can also reach outside of their scientific circles through use of popular hashtags. Hashtags enhance the visibility of an Instagram post as it then becomes searchable and may even be featured in Instagram’s dynamic “top” or featured lists of posts for that hashtag. Strategic use of hashtags that resonate with key target demographics can help an Instagrammer get their topic seen by broader audiences who follow that hashtag but not the Instagrammer him- or herself [64]. For example, a mix of science-specific hashtags and less science-focused hashtags will allow an Instagram post to perform well but also show up in the feeds of those who may not regularly follow science content. This is a common technique used in digital marketing that depends on some level of prior “audience listening” to anticipate popular trends and key words.

In summary, the growth and visibility of a diverse, humanized scientific community on IG begs a greater understanding of how viewers evaluate scientist Instagrammers and whether connecting with scientists on IG fosters trust. This study sought to fill a gap in understanding how humanized content posted by scientists on Instagram impacts perceptions of scientists among viewers. We were interested to see whether such humanized content could improve perceptions of scientists’ warmth, as suggested by prior studies on the impacts of positive facial expressions on social cognition, and change stereotypes about what a scientist typically looks like, or the typical characteristics and demographics of scientists.

## Research questions and hypotheses

Our primary research question for this study was whether scientist selfies on IG posted by scientist Instagrammers (IGers) change stereotypes of scientists’ warmth vs. competence. We expected that individuating a multitude of scientists in selfies displaying warmth traits (broad smiles, eye contact, friendly gestures) would help change group warmth stereotypes for scientists in general. Based on our literature review, we hypothesized the following:

H1 Scientist IGers who post selfies will be perceived as warmer than scientist IGers who post science-only photos.
H2 Scientist selfies on IG will positively influence perceptions of scientists’ warmth in general, as compared to science-only IG posts.

We also explored the impact of stimulus condition on competence, although we did not expect scientists posting selfies to be perceived as more or less competent than scientists posting science-only photos. We did not expect changes in perceived competence for our IGers or scientists in general given the presence of prominent scientific competence cues (lab coats, scientific equipment, etc.) and scientific captions used consistently across stimulus groups.

We predicted that female scientists would be perceived as warmer than male scientists. We also expected that a strong representation of women scientists in IG posts would positively influence perceptions of gender science stereotypes as well as perceptions of scientists’ warmth.

H3a Female scientist IGers will be perceived as warmer than male scientist IGers.
H3b Female scientist IG posts (especially selfies) will result in positive changes in gender science stereotypes, as compared to male scientist IG posts, especially for selfies.
H3c Female scientist IG posts (especially selfies) will result in more positive perceptions of scientists’ warmth in general, as compared to male scientist IG posts.

## Materials and methods

### Online survey experiment

This study was approved by the Louisiana State University Institutional Review Board: IRB# E10449, Expiration 4/12/2020. Participants agreed to participate in the study consent by viewing an electronic consent form—written consent was not required (data analyzed anonymously). Survey respondents were recruited and screened through a paid Qualtrics Panel to meet U.S. representative quotas on gender, education and age (see SI). We worked with Qualtrics to eliminate and replace 200 “trash” or unintelligible survey responses based on an open-ended stimulus recall question. Respondents viewed IG content embedded in a private Squarespace website designed to look like an aggregation site for posts from a “Scientists of Instagram” Rocur IG account (see www.scientistsofig.com). Participants were instructed to return to our survey to complete the study after browsing the content to answer questions about the IGers they encountered.

#### Data processing

A total of 1,620 survey responses were recorded and included in our final analyses. These included complete and partial but substantially complete responses. We removed responses where the stimulus materials had been viewed for less than 15 seconds. Where we detected that respondents had entered “5” or “1” across the board on warmth/competence scales, regardless of survey item direction, we removed these responses. Following this removal, no extreme outliers for warmth/competence were detected in SPSS (interquartile range test outlier test). Our final analyses were based on 339 respondents viewing control IG posts, 319 respondents viewing science-only male posts, 313 respondents viewing science-only female posts, 318 respondents viewing male selfies, and 329 respondents viewing female selfies. Our SPSS dataset (S1 Dataset) is available online with a filter variable labeled “Outlier does not equal 1 (FILTER)” provided to remove the inappropriate responses as described above.

### Experimental design and stimulus content

All research participants were randomly assigned to one of five different experimental conditions, in all of which they saw a series of Instagram posts (photographs and captions): (1) a control IG account of male and female “Humans of Southeastern” (@humansofselu, lab experiment) or “Humans of Broadway” (@humansofbroadway, online survey experiment); (2) a “Scientists of IG” account depicting seven photos of scientific objects / equipment in lab or field settings, posted by seven different male scientists as identified by first name / pseudonym in the caption (Sam, Liam, Deboki, Martin, Kyle, Luis, Ian); (3) same as (2) but posted by seven different female scientists (Samantha, Laura, Deboki, Martha, Kylla, Luisa, Imogene); (4) a “Scientists of IG” account depicting seven photos of the same scientific objects / equipment but with a male scientist’s smiling face present; (5) same as (4) but with all female faces. We refer to these stimulus conditions throughout this manuscript as: (1) control; (2) science-only male; (3) science-only female; (4) male selfies; (5) female selfies. Selfies were recreations of science-only photos (same objects/equipment and same overall scene) but containing a scientist’s smiling face. For example, one science-only photo depicted a colorful bioreactor set up on a lab bench, while the selfie equivalent depicted the bioreactor from the same angle but with a male/female scientist in the shot pointing to the equipment and smiling at the camera. See S1 Appendix for a detailed description of stimulus content.

Following stimulus exposure, participants were asked questions about their enjoyment of the content and their impressions of the IGers they had encountered. They were then asked 1) to evaluate the warmth and competence of individual IGers as represented by a select sample of visual-only screenshots from the stimulus they had just browsed (embedded in an online questionnaire), and 2) to answer questions about their perceptions of scientists in general. We randomized the order of these question sections (IGer evaluations and scientist stereotypes) for our online survey experiment.

**Fig 1:**
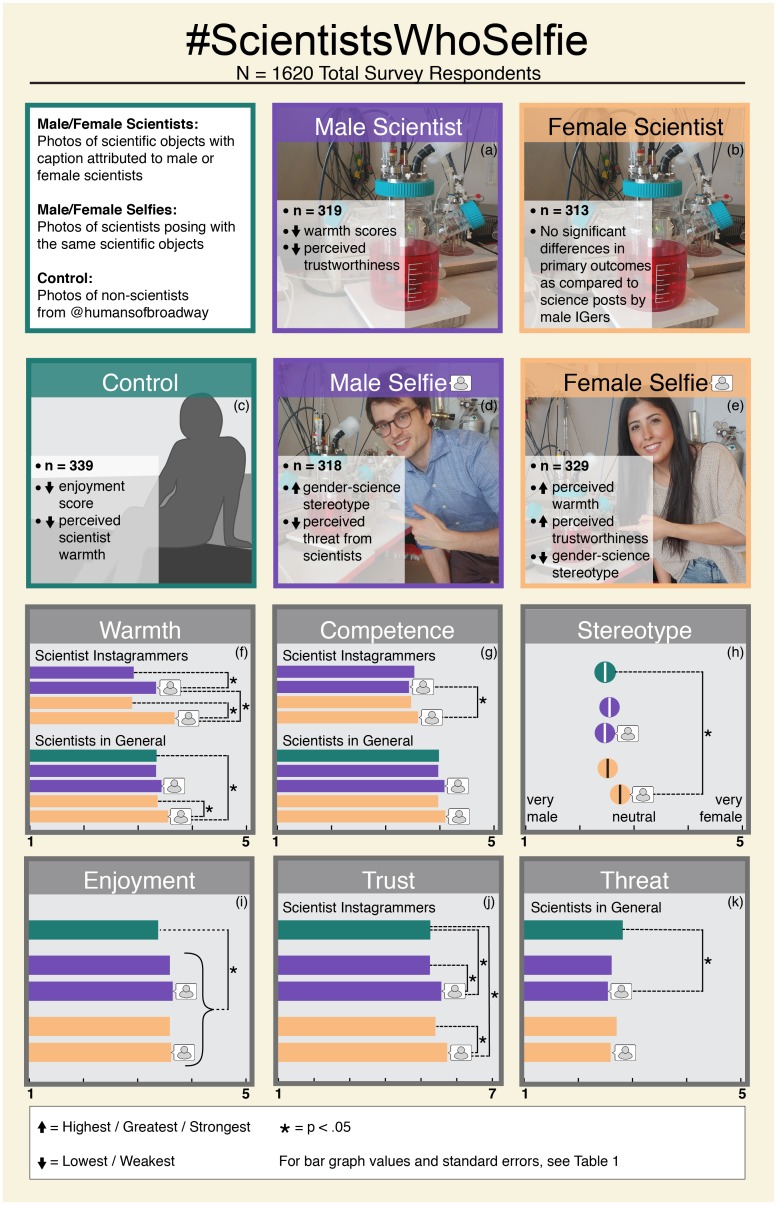
Scientists selfie example images and outcome means by stimulus group. Bar graphs (boxes “f”: through “l”) represent impacts of IG stimulus viewing on enjoyment (box i), perceived trustworthiness of the IGers (box j), perceived symbolic threat from scientists (box k), warmth and competence evaluations of individual scientist IGers (top of boxes f and g), perceived warmth and competence of scientists (bottom of boxes f and g), and gender science stereotypes (box k). For bar graph values and standard errors, see Table 1. Asterisks (*) denote significant differences between means (per box) connected by dotted lines. Select comparisons are represented visually for emphasis; for an exhaustive list, see Table 1. Figure created by Jen Burgess, Isoline Studios.

We complied with the terms of service for the sites and social media platforms that we used in this study for stimulus exposure, including Instagram and Squarespace. We provided accurate information about the scientific work of the scientists featured in our stimulus images, with their permission, using their first names / pseudonyms in the IG post captions (pseudonyms created so that the male and female names for corresponding male and female selfies were complimentary, e.g. Sam and Samantha). All stimulus content (images and captions) was created originally for this study and used with the permission of the creators. All individuals who helped create content and who were featured in the stimulus images for our study, some of which are featured in Fig 1, have given written informed consent (as outlined in the PLOS consent form) to publish these case details, their real names and the photographs/self-portraits they produced for our study.

### Measures

See S1 Appendix for all survey question wording and more information on scale items.

#### Enjoyment

We asked respondents to rate their enjoyment of the stimulus content in a series of questions about how interesting, visually appealing, credible, useful, and educational the IG posts were. These questions were combined into a 9-item enjoyment scale (Cronbach’s alpha = .93, *M* = 3.57, *SD* = .82).

#### Perceptions of scientist IGers

We measured how trustworthy respondents perceived the IGers portrayed in stimulus content to be on 7-point Likert scale. For the selfie conditions we also measured the extent to which respondents thought the IGers looked like scientists (5-point Likert-type scale). We measured the extent to which respondents saw themselves as similar or dissimilar to the IGers in terms of values, goals, opinions, thoughts, and beliefs (7-point bipolar scales based on opposite word pairs). These questions were combined into a 5-item value similarity scale (Cronbach’s alpha = .89, *M* = 4.17, *SD* = 1.36).

#### Warmth and competence of scientist IGers

We used a selection of four photos from our experimental IG posts to ask respondents to evaluate the warmth, competence, and physical attractiveness of individual scientist IGers. Based on photos without captions, respondents were asked how warm/competent/attractive the person who took each photo (science-only posts) or the person in each photo (selfie posts) was, on a 5-point scale from not at all to extremely.

#### Warmth and competence stereotypes of scientists

We used a question derived from Fiske’s work on stereotypes of scientists [12] to measure the warmth and competence stereotypes. We told survey respondents that we were interested in learning how different groups of people are viewed by society, and asked them to indicate on scale from 1 (not at all) to 5 (extremely) how well a range of different words represent traits of scientists, in general (*Generally speaking, scientists are…*). Respondents rated 20 different words for how well they represent traits of scientists. Included were words representing warmth (Cronbach’s alpha = .90, *M* = 3.37, *SD* = .65; 9 items: sincere, honest, warm, helpful, sociable, ethical, likeable, friendly, trustworthy) and competence (Cronbach’s alpha = .78, *M* = 4.01, *SD* = .71; 3 items: competent, confident, intelligent).

#### Explicit gender science stereotypes

We asked respondents how much they associated different activities, including science, liberal arts, and social media use, with being male or female, on a 5-point Likert-type scale from Strongly Male to Strongly Female.

#### Media habits and science interest

We measured respondents’ frequency of seeking out science information online, frequency of social media and IG use. We asked respondents to rate their interest in information about a range of topics including: science, politics, sports, business and finance, and health and medicine. We asked respondents whether they knew a scientist personally and to name one or more.

#### Demographics

We asked survey respondents to self-report sex, age, ethnicity, level of education, degree field (if at least a 2-year degree), number of science classes taken in college, political affiliation, and importance of religion in their lives.

### Statistical analysis

We used SPSS to analyze survey responses. For hypothesis testing, we used ANOVA/MANCOVA tests with Bonferroni-adjusted post hoc analyses and linear regression models.

## Results

### Online survey experiment

In an online survey experiment, participants (N = 1620) browsed a total of seven control or scientist-produced IG posts (either science-only posts created by male scientists, science-only posts created by female scientists, selfie posts of male scientists, or selfie posts of female scientists). These IG posts were originally tested within a lab environment in a pilot study among 162 undergraduate students (see S1 Appendix and Table A in S1 Appendix for methods and results). We found in our pilot study that scientists in selfies were perceived as significantly warmer than scientists posting science-only images.

### Demographics

A total of 891 respondents or 55% of the final sample identified as female, with a mean age of 46 years (*SD* = 16.6, range = 85 years). Participants primarily self-identified as White (77%), Black (9%), or Hispanic/Latino (6%). Roughly 30% of respondents had attained at least a Bachelor’s (4 year) degree, with only 10% having a degree in a STEM field. A majority of the respondents were social media users (87%, n = 1408) and nearly half (46%, n = 747) indicated having an IG account. See other demographic information in Table B in S1 Appendix.

### Descriptives

Respondents generally indicated having similar levels of interest in information about science (*M* = 3.1, *SD* = 1.2) as in politics (*M* = 3.2, *SD* = 1.3), sports (*M* = 3.0, *SD* = 1.4), and business (*M* = 3.0, *SD* = 1.2), with the highest interest in health and medicine (*M* = 3.5, *SD* = 1.1). Surprisingly, 32% (n = 433) of social media users indicated following at least one scientist. However, only 18% of all respondents (n = 284) indicated knowing a scientist personally. Overall, respondents perceived scientists in accordance with cultural stereotypes. Based on our control group (n = 339), scientists are seen as highly competent (*M* = 4.00, *SD* = .68), but only moderately warm (*M* = 3.29, *SD* = .61). Respondents in the control group also tended to relate science more with being male than with being female (*M* = 2.45 on a 5-point scale from strongly male to strongly female, *SD* = .87), but liberal arts (*M* = 3.34, *SD* = .79) and social media use (*M* = 3.43, *SD* = .86) more with being female. When asked about the IG content they had seen during stimulus exposure, respondents generally indicated having enjoyed it (*M* = 3.56 on a 5-point scale, *SD* = .82). Respondents generally perceived the IGers they had encountered to be trustworthy (*M* = 5.35 on a 7-point scale, *SD* = 1.21).

### Impacts of stimulus exposure

We investigated overall impacts of stimulus exposure on enjoyment; perceived trustworthiness, warmth, and competence of the encountered IGers; warmth and competence stereotypes of scientists; perceived symbolic threat of scientists; and gender science stereotypes. We did so in a series of ANCOVA analyses controlling for participant gender, age, education, interest in science, IG use, importance of religion, and knowing a scientist personally. We used Bonferonni-adjusted post hoc comparisons to investigate differences on key outcome variables between each of the stimulus conditions. See results in Table 1 and Fig 1.

**Table 1:** Impacts of stimulus on key outcomes—ANCOVA analyses with post hoc estimated mean contrasts by stimulus group.

	Estimated marginal mean (standard error)						
	Control	Science Male	Science Female	Selfie Male	Selfie Female	*F*	*η*_2_
Enjoyment	3.38(.04)_*a*_	3.59(.04)_*b*_	3.61(.04)_*b*_	3.65(.04)_*b*_	3.63(.04)_*b*_	6.82***	.017
IGers							
Warmth^1^		2.91(.04)_*a*_	2.88(.04)_*a*_	3.33(.04)_*b*_	3.67(.04)_*c*_	76.67***	.157
Competence^1^		3.57(.05)_*ab*_	3.51(.05)_*ab*_	3.47(.05)_*a*_	3.64(.04)_*b*_	2.82*	.007
Warmth^2^		3.00 (.04)_*a*_	2.92(.04)_*a*_	3.39(.04)_*b*_	3.49(.04)_*b*_	48.44***	.105
Competence^2^		3.65(.04)_*a*_	3.55(.04)_*ab*_	3.52(.04)_*ab*_	3.48(.04)_*b*_	2.90*	.007
Trust	5.17(.06)_*a*_	5.16(.07)_*a*_	5.31(.07)_*ab*_	5.47(.07)_*bc*_	5.63(.07)_*c*_	9.60***	.024
Scientists							
Warmth	3.31(.04)_*a*_	3.30(.04)_*a*_	3.33(.04)_*a*_	3.40(.04)_*ab*_	3.52(.04)_*b*_	6.62***	.017
Competence	3.97(.04)_*a*_	3.96(.04)_*a*_	3.96(.04)_*a*_	4.07(.04)_*a*_	4.09(.04)_*a*_	2.47*	.006
Threat	2.77(.06)_*a*_	2.57(.06)_*ab*_	2.66(.06)_*ab*_	2.50(.06)_*b*_	2.55(.06)_*ab*_	3.22*	.008
Gender Stereotypes	2.44(.04)_*a*_	2.51(.05)_*a*_	2.48(.05)_*a*_	2.42(.05)_*a*_	2.70(.04)_*b*_	6.20***	.016
Looks Like Scientists	-	-	-	3.30(.06)	3.30(.96)	.01	.00

*Notes*: Results based on SPSS GLM ANCOVA analyses. Degrees of freedom are *F*(4, 1569) for Enjoyment, Trust, Warmth/Competence, Gender Stereotypes; *F*(4, 1568) for Threat; *F*(3, 1237) for IGer Warmth/Competence; *F*(1, 623) for Look Like Scientists. For models of IGer warmth/competence denoted with a “2” superscript, perceived attractiveness was added as a covariate. Means with differing subscripts within rows are significantly different at or below the p < .05 based on Bonferroni post hoc pairwise comparisons.

*p < .05.

**p < .01.

***p < .001.

#### Perceptions of Instagrammers

There was a significant main effect for stimulus on enjoyment (across all conditions: Stimulus *F*(4, 1569) = 6.82, *p* < .001; Model *F*(11, 1569) = 23.10, *p* < .001). Participants who had browsed any experimental science IG posts enjoyed the content significantly more than did participants who had viewed control IG posts (*p* < .01). There was also a main effect of stimulus on the perceived trustworthiness of the IGers (Stimulus *F*(4, 1569) = 9.60, *p* < .001; Model *F*(11, 1569) = 13.58, *p* < .001)). Participants who had browsed selfies, especially those containing a female scientist’s face, perceived the IGers they had encountered to be significantly more trustworthy than did participants who had browsed control or science-only posts (*p* < .001).

There was also a main effect of stimulus on the perceived warmth (Stimulus *F*(3, 1237) = 76.67, *p* < .001, Model *F*(10, 1237) = 29.67, *p* < .001) and competence (Stimulus *F*(3, 1237) = 2.82, *p* < .05, Model *F*(10, 1237) = 9.15, *p* < .001) of the IGers. When asked to evaluate the warmth (single item) of select scientists from our IG posts, participants viewing selfies evaluated the IGers, on average, as significantly warmer than did participants viewing science-only photos (*p* < .001). Participants viewing female selfies evaluated the IGers as significantly warmer than participants viewing male selfies (*p* < .001). For competence (single item), few significant differences were observed between stimulus conditions apart from female IGers in selfies receiving higher competence evaluations than male IGers in selfies (*p* < .05).

#### Science stereotypes

We found a significant impact of stimulus on warmth of scientists (across all conditions: *F*(4, 1569) = 6.62, *p* < .001; Model *F*(11, 1569) = 11.18, *p* < .001). Participants who had browsed selfies of either gender, but particularly female selfies, evaluated scientists as warmer than did participants who had browsed control (*p* < .001) or science-only IG posts (*p* < .01). These results are based on an ANCOVA analysis with a collapsed stimulus variable (control vs science-only vs selfies, *F*(2, 1571) = 10.46, *p* < .001). Participants who had viewed selfies also evaluated scientists as more competent overall (across all conditions: *F*(4, 1569) = 2.48, *p* < .01; Model *F*(11, 1569) = 8.07, *p* < .001) and perceived less threat from scientists (*F*(4, 1568) = 3.22, *p* < .05; Model *F*(11, 1568) = 14.35, *p* < .001). Perceived threat was lowest when participants had browsed selfies from scientists of the opposite sex. The estimated marginal means and standard errors (*M*(*SE*)) for threat are: 2.83(.09) for control, 2.48(.09) for female selfies and 2.56(.08) for male selfies viewed by men (no significant means comparisons); 2.75(.08) for control, 2.42(.09) for male selfies and 2.60(.08) for female selfies viewed by women (only significant mean comparison is between male selfies and control, *p* < .05).

### Predictors of Instagrammer warmth and competence

We hypothesized that scientist IGers in selfies would be perceived as warmer than scientist IGers posting science-only photos (H1). We ran linear regression models to identify predictors of individual IGers’ average perceived warmth (single item; *F*(12, 1235) = 48.64, *p* < .001; R2 = .32) as well as competence (single item; *F*(12, 1235) = 29.58, *p* < .001; R2 = .22). We found stimulus (science-only versus selfie) to be a significant predictor of scientist IGer warmth (*β* = .35, *p* < .001) but not competence (*β* = -.01, *p* = .88). We accounted for scientist gender, enjoyment, participant age, gender, education, interest in science, importance of religion, IG use, political affiliation, and knowing a scientist personally. IGers in selfies were evaluated as significantly warmer than IGers posting science-only photos. There were no significant differences in perceived competence. Scientist gender was a significant predictor of warmth (*β* = .09, *p* < .001) but not competence. Female scientists received the highest warmth evaluations. These results support H1, that scientists in selfies are perceived as warmer than scientists posting science-only photos on IG, and H3a. Other predictors of scientist IGer warmth and competence evaluations are enjoyment and participant IG use. For perceived competence of the scientist IGers, participant education level was a significant predictor. See regression model results in Table 2.

**Table 2:** Linear regression analysis predicting scientist Instagrammer warmth and competence.

Model 1	Warmth		Competence	
	*β*	95% CI of *B*	*β*	95% CI of *B*
Constant		[.65, 1.22]		[1.26, 1.86]
Stimulus	.35***	[.51, .66]	-.01	[-.09, .08]
Scientist gender	.09***	[.08, .24]	.03	[-.03, .14]
Enjoyment	.40***	[.35, .45]	.42***	[.36, .47]
Participant gender	.04	[-.01, .15]	.04	[-.03, .14]
Participant age	-.01	[-.01, .01]	.01	[-.01, .01]
Participant education	.03	[-.03, .05]	.08**	[.01, .08]
Interest in science	.04	[-.01, .07]	.04	[-.01, .08]
Religion	.04	[-.01, .05]	.02	[-.02, .04]
Know a scientist	-.04	[-.20, .02]	.01	[-.10, .12]
Instagram use	.08**	[.01, .06]	.06*	[.01, .05]
Democrat vs Other	.04	[-.03, .18]	.05	[-.02, .20]
Indep. vs Other	.02	[-.06, .13]	-.01	[-.11, .09]
Attractiveness (Model 2)	.31**	[.33, .46]	.26***	[.26, .40]
*F* total	48.64***		29.58***	
R^2^	.32		.22	

Notes: *β* = standardized coefficient. B = unstandardized coefficient. CI = confidence interval. Degrees of freedom for both regression equations are *F*(12, 1235). Stimulus variable represents science-only posts versus selfie posts. Only weak correlations are found between predictors: IGer attractiveness and scientist gender are weakly correlated (Pearson coefficient = .23, p < .001), as are IG use and age (Pearson coefficient = -.36, p < .01). Gender variables are coded as Male (0) vs Female (1).

*p < .05.

**p < .01.

***p < .001.

When we added perceived attractiveness (an average of attractiveness evaluations) of the IGers to our linear regression models, we found that attractiveness strongly predicted both perceived competence and warmth. Using Andrew Hayes’ PROCESS version 3.1 (Model 4) in SPSS [65], we found that attractiveness mediates effects of scientist gender on warmth evaluations (Total effect = .16, *t*(1235) = 3.95, *p* < .001; Direct effect = .04, *t*(1234) = 1.0, *p* = .32; Indirect effect = .12). Attractiveness also mediates effects of stimulus on competence evaluations. Female scientists in selfies, evaluated as highly attractive, are perceived as more competent than male scientists, but not when controlling for attractiveness (see Table 1).

### Predicting Instagrammer trustworthiness

We also ran a linear regression model predicting perceived trustworthiness of the encountered IGers (*F*(11, 1569) = 15.26, *p* < .001; R2 = .10). See Table D in S1 Appendix. We measured perceived trustworthiness after stimulus exposure and before participants evaluated the warmth and competence of select individual IGers. We found that participants who had browsed selfies evaluated the IGers they had encountered as significantly more trustworthy than participants who had browsed science-only (*β* = .12, *p* < .001) or control IG posts (*β* = .13, *p* < .001). Other significant predictors included age, IG use, and political affiliation. Participants who were younger (*β* = .05, *p* < .05), more interested in science (*β* = .22, *p* < .001), more frequent IG users (*β* = .09, *p* < .01), and Democrats (versus Republicans; *β* = .12, *p* < .001) evaluated the scientist IGers as more trustworthy. When we added scientist gender into the model (control group excluded), we found that participants who had browsed female scientist IG posts evaluated the IGers as significantly more trustworthy than did participants who had browsed male scientist IG posts (*β* = .06, *p* < .05; *F*(11, 1236) = 12.81, *p* < .001; R2 = .10). Results for the other predictors remained consistent.

### Predicting science stereotypes

We hypothesized that seeing selfies of scientists, via processes of individuating scientists and evaluating their smiling faces, would positively influence stereotypes of scientists’ warmth (H2). We ran linear regression models predicting perceptions of scientists’ warmth (*F*(11, 1569) = 13.01, *p* < .001; R2 = .08) as well as competence (*F*(11, 1569) = 8.71, *p* < .001; R2 = .06). See results in Table 3, Model 1. We accounted for factors that could impact stereotypes including participant age, gender, education, interest in science, importance of religion, IG use, political affiliation, and knowing a scientist personally. Two dummy coded stimulus variables, 1) control versus other and 2) science-only versus other (reference group was selfies, male and female), were significant predictors of both warmth and competence. Participants who had browsed selfies perceived scientists in general to be both warmer and more competent than participants who had browsed control or science-only IG posts. We thus found support for H2; scientist selfies positively influenced perceptions of scientists’ warmth over science-only IG posts by scientists. We did not expect the increase in perceived competence that scientists received from participants who had browsed selfies, although additional competence cues (e.g. scientists working in the lab) and attractiveness of selfies could have played a role.

**Table 3:** Linear regression analysis predicting scientist warmth and competence stereotypes.

Model 1	Warmth		Competence	
	*β*	95% CI of *B*	*β*	95% CI of *B*
Constant		[1.69, 3.10]		[3.17, 3.62]
Selfie vs Control	.09**	[.07, .23]	.06*	[-.20, -.01]
Selfie vs Science	.10***	[.07, .21]	.07**	[-.19, -.04]
Participant gender	.03	[-.03, .10]	.06*	[.02, .16]
Participant age	.03	[-.01, .01]	.08**	[.001, .01]
Participant education	.-03	[-.04, .01]	.06*	[.01, .06]
Interest in science	.22***	[.09, .15]	.18***	[.07, .13]
Religion	.01	[-.02, .03]	-.02	[-.03, .02]
Know a scientist	-.03	[-.14, .03]	-.03	[-.16, .03]
Instagram use	.09**	[.01, .05]	.04	[-.01, .03]
Democrat vs Other	.11***	[.08, .24]	.03	[-.05, .13]
Indep. vs Other	-.01	[-.10, .06]	-.05	[-.16, .02]
Scientist Gender (Model 2)	.05 (p= .08)	[-.01, .14]	.01	[-.08, .08]
*F* total	13.01***		8.71***	
R^2^	.08		.06	

*Notes*: *β* = standardized coefficient. B = unstandardized regression coefficient. CI = confidence interval. Degrees of freedom for Model 1 regression equations are F(11, 1569) for Warmth/Competence. Only weak correlations are found between predictors: IG use and age (Pearson coefficient = -.36, p < .01). When we include enjoyment as a predictor (β = .36, p < .01), the significance of the selfie vs. control variable is affected (p = .07), but not the significance of the selfie vs. science variable. Dummy variables are coded as X (1) vs Other (0). Gender variables are coded as Male (0) vs Female (1).

*p < .05.

**p < .01.

***p < .001.

#### Individual scientist evaluations as mediators of stereotypes

**Fig 2:**
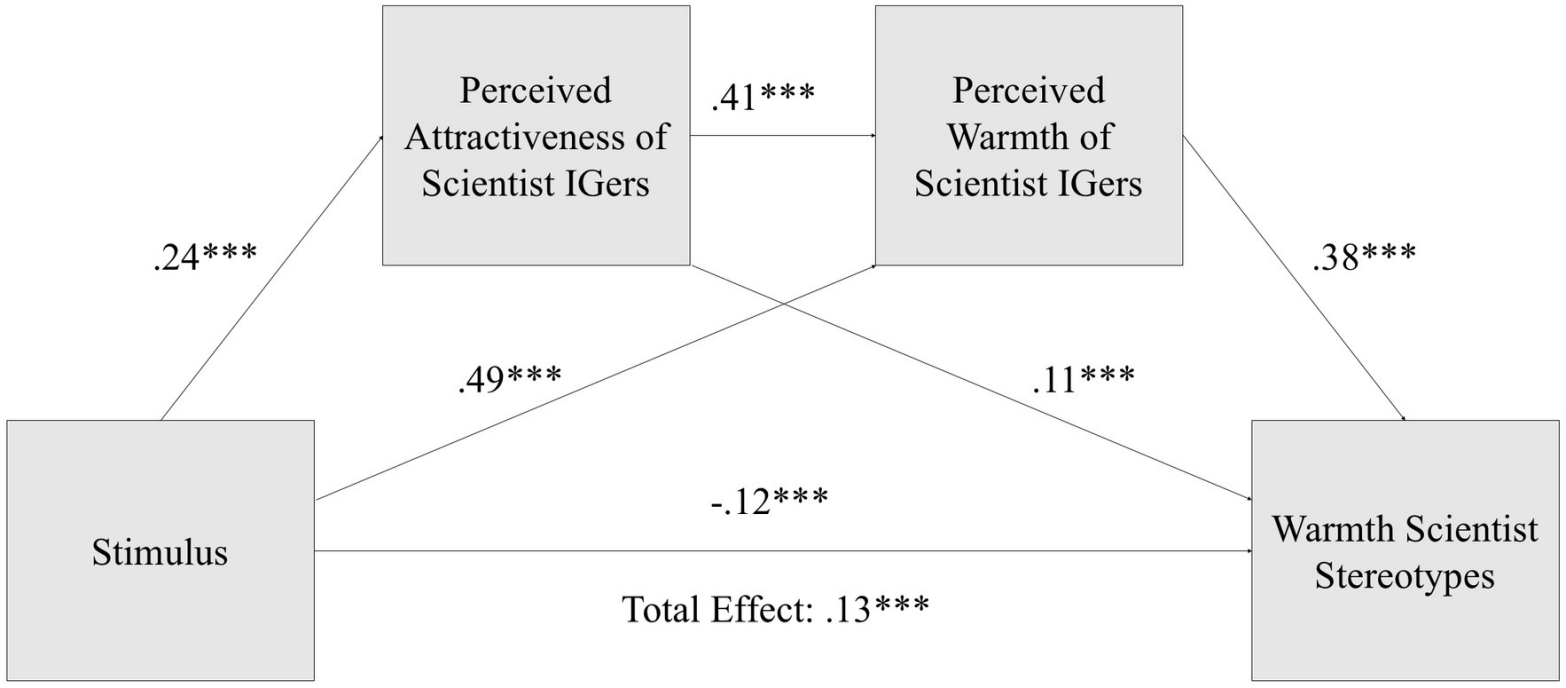
Mediation model for direct and indirect effects of stimulus on warmth stereotypes of scientists.

We explored whether attractiveness and warmth evaluations of individual IGers were mediators of stimulus effects on stereotypes. Using Hayes’ PROCESS version 3.1 (Model 6) in SPSS [65], we found that perceived attractiveness and warmth of individual IGers fully mediate the impact of stimulus (science-only vs. selfies) on warmth stereotypes of scientists. See Fig 2. We added the following as covariates: participant gender, age, education, interest in science, knowing a scientist personally, political affiliation, IG use, and enjoyment. Scientists in selfies were evaluated as more attractive (*β* = .24, t(1236) = 6.93, *p* < .001; Model R2 = .16, *p* < .001) and warmer (*β* = .49, t(1235) = 12.84, *p* < .001; Model R2 = .40, *p* < .001) than scientists posting science-only photos. Warmth stereotypes of scientists in general were also predicted by both perceived attractiveness (*β* = .11, *t*(1234) = 4.00, *p* < .001; Model R2 = .40, *p* < .001) and perceived warmth of individual IGers (*β* = .38, *t*(1234) = 16.70, *p* < .001). Perceived attractiveness was a significant predictor of warmth in evaluations of individual IGers (*β* = .41, *t*(1235) = 12.83, *p* < .001). Thus there were the following significant indirect effects: Stimulus > attractiveness > stereotypes (Effect = .03, 95% CI [.01, .04]); stimulus > warmth of IGers > stereotypes (Effect = .18, 95%CI [.14, .23]); stimulus > attractiveness > warmth of IGers > stereotypes (Effect = .04, 95% CI[.02, .05]). The total indirect effect of stimulus on warmth stereotypes (Effect = .25, 95%CI [.20, .29]) is greater than the total effect (*β* = .13, *t*(1236) = 3.81, *p* < .001). The stimulus condition combines selfies of both genders. However, female scientists in selfies are evaluated as significantly more attractive than male scientists (*p* < .001), and male scientists in selfies are not evaluated as any more attractive than the scientists who take science-only photos are guessed to be (see SI).

We also found that attractiveness of IGers is a mediator of stimulus effect on competence stereotypes in a similar PROCESS (Model 4) analysis with covariates as described above. Without attractiveness, stimulus had a significant and positive total effect on competence stereotypes (*β* = .10, t(1236) = 2.69, *p* < .01; Model R2 = .17, *p* < .001). However, attractiveness was also a significant predictor of competence stereotypes (*β* = .14, *t*(1235) = 4.54, *p* < .001; Model R2 = .19, *p* < .001) and suppressed the direct impact of stimulus on competence when included in the regression model (*p* = .07).

### Gender science stereotypes and female scientists’ warmth and competence

We hypothesized that seeing female scientists in IG posts would counteract gender science stereotypes and promote warmth (H3b and H3c). We ran ANCOVA analyses and linear regression models using dichotomous factors for stimulus condition (science-only vs. selfies) and scientist gender (male vs. female). We also accounted for participant age, gender, education, interest in science, importance of religion, IG use, political affiliation, and knowing a scientist personally.

We found that only female scientist selfies resulted in a significant shift in stereotypes that associate STEM fields with being male (see Table 2). In a linear regression model (*F*(11, 1236) = 5.36, *p* < .001; R2 = .04), the presence of female scientists in IG posts was a significant predictor (*β* = .08, *p* < .01) of female-shifted science stereotypes, while overall stimulus condition (science-only vs. selfies) was not a significant predictor (*β* = .04, *p* = .21). Other significant predictors of female-shifted stereotypes were younger age (*β* = -.08, *p* < .05), lower education level (*β* = -.15, *p* < .001), and less IG use (*β* = -.07, *p* < .05) among participants. Thus, we find support for H3b.

To investigate warmth stereotypes by scientist gender, we ran a linear regression model (*F*(11, 1236) = 10.23, *p* < .001; R2 = .08) predicting warmth using two variables: a collapsed stimulus variable (science-only vs. selfies) and a scientist gender variable (male vs. female) (see Table 3, Model 2). All other predictors included in the model were the same as those used to investigate warmth in the Predicting Science Stereotypes section above. We found that across all stimulus conditions, scientist gender was not a significant predictor of warmth. However, when we used dummy coded variables for all stimulus conditions with the control group as the reference (*F*(13, 1567) = 11.38, *p* < .001; R2 = .09), we found that only female scientist selfies resulted in significantly greater perceptions of scientists’ warmth (*β* = .12, *p* < .001). Male scientist selfies resulted in marginally greater perceptions of scientists’ warmth compared to control images (*β* = .06, *p* < .06). Female scientist selfies resulted in significantly greater perceptions of scientists’ warmth compared to every other stimulus condition individually (vs. sci male, *β* = .12, *p* < .001; vs. sci female, *β* = .11, *p* < .001; vs. selfie male, *β* = .06, *p* < .04). This increase in perceived warmth was accompanied by an increase in perceived competence. Only female scientist selfies resulted in significantly greater perceptions of scientists’ competence in general (*F*(13, 1567) = 7.37, *p* < .001; R2 = .06; *β* = .06, *p* < .05), with the control group as reference.

## Discussion

Scientists’ less-than-warm public image is problematic because warmth is a key component of trust [19]. To build trust, scientists might consider humanizing science through friendly self-portraiture in scientific settings. Scientists are generally perceived as competent but often stereotyped as nerdy and socially inept, or more generally not warm. We found that humanizing scientists via Instagram selfies can counter these stereotypes.

Viewers of scientist selfies, both from male and female scientists, perceived both scientist Instagrammers and scientists in general to be warmer and at least as competent or more competent than viewers of science-only or control Instagram posts did. Seeing scientist selfies—as opposed to images of scientific objects posted by scientists online—thus appears to selectively reduce negative stereotypes, likely through a process of individuating scientists based on their faces. Here, this process involved seeing a series of smiling faces of particularly counterstereotypic scientists and identifying them as “warm” individuals as opposed to unfamiliar members of an envied and relatively “cold” outgroup. In terms of preserving perceived competence, competence cues (e.g. a scientific setting, scientific equipment, recounting of scientific work being conducted visually and through photo captions), may be an important feature of scientist selfies.

We found gender to be a major driver of changes in stereotypes of scientists’ warmth and gender science stereotypes. We found that seeing a series of female scientist selfies on Instagram can shift stereotypes that associate STEM fields with being male, as well as positively shift stereotypes about scientists’ warmth. Seeing and evaluating individual smiling/friendly faces contributed to this warmth shift. These findings again suggest that people viewing scientists’ Instagram posts begin to individuate scientists as opposed to categorizing them into an outgroup that is competent but cold. This individuating process could also help explain why female scientist IGers posting selfies were not evaluated as stereotypically less competent than male scientist IGers, even if their gender elicited greater perceived warmth. Their identity as competent scientists may have held a prominent sway over these evaluations. Counter to our initial concerns that female scientist selfies could trigger gender stereotypes causing viewers to perceive these scientists as warmer but also less competent and more narcissistic, for example, we found that female scientist selfies actually led to more positive competence evaluations for scientists overall and significantly reduced perceptions that scientists in general are “vain” (see SI).

Although previous research has shown that female smiling faces may influence men’s social judgments to a larger extent than women’s social judgments [55], we found that our female scientist selfies had a similar effect for both male and female viewers. On the other hand, science-only photos posted by female scientists did not significantly shift gender science stereotypes. This could be because the female names in the captions were not attention-grabbing and/or memorable, or because participants did not read the captions. We did not evaluate whether participants properly attributed our science-only posts to scientists of the intended gender. However, it is likely that female scientist selfies were most successful in moving gender science stereotypes due to the greater accessibility of gender in these posts.

In summary, we suggest that humanizing, visual science communication may be one way for broader audiences to “get to know” scientists. However, we believe that scientists should be genuine and transparent in humanizing themselves. By sharing selfies and stories from their daily lives in the lab/field, talking about their motivations and struggles, inviting viewer participation, and opening up the scientific process, scientists could foster trust by individually and collectively embodying warmth as well as competence.

Social media platforms like Instagram are unique in the opportunities they offer researchers to reach public audiences. Social media platforms give diverse researchers a platform to express themselves freely—an opportunity traditional media outlets historically have not given women and minorities [66]. Social media platforms could represent a turning of the tide for perceptions of scientists if leveraged by diverse individuals to interact with broad audiences in ways that communicate warmth and build mutual trust. We acknowledge that reaching truly broad audiences can be a challenge for the scientist communicator. Training and planning can help. For example, the Instagram algorithm relies on engagement to evaluate which posts users see in their feed. The strategic use of relevant hashtags and interactive options (e.g. quizzes and fill in the blank prompts unique to the Instagram Story) are likely to increase overall engagement rates and thus increase scientists’ exposure to non-scientist audiences.

### Limitations

We cannot pinpoint the exact mechanism by which selfies increased participants’ perceived warmth of scientist Instagrammers and changed their stereotypes of scientists. We prioritized externally valid stimulus content (created for us by scientists in the IG community) and guided participants to browse a series of Instagram posts that combined multiple warmth-related characteristics. For example, selfie conditions consisted of seven smiling scientist faces of different ethnicities and styles, together with captions written from the perspective of each featured scientist. The presence of smiling expressions, the diversity in scientist appearance (including ethnicity), and the combination of selfies with captions describing scientists’ motivations to do their work—“I have been developing new and exciting technologies using genetic engineering to make our immune system better at fighting cancer”—might all have contributed to changes in stereotypes. The effects of these stimulus elements should be teased apart in future work.

The physical attractiveness of our scientist Instagrammers also played a role in their perceived warmth and competence. We observed that attractiveness is a mediator of individual competence and warmth evaluations. Our female scientist Instagrammers were consistently evaluated as more attractive than male scientist Instagrammers, although this could be due to stereotypes that focus on women’s physical appearance and lead to female smiling faces being more positively evaluated than male smiling faces. We did not manipulate but rather measured attractiveness in our experiments. That said, we note that we tried to match the physical appearance of male and female scientists in selfies (similar clothing, only light makeup, etc.).

Perceived attractiveness has been associated with assessments of moral goodness, aka the “Beauty-Is-Good” stereotype [67], trustworthiness [68], and intelligence [69]. This may help explain the boosts in competence and warmth that our female scientist Instagrammers in selfies received. The Beauty-Is-Good stereotype particularly influences judgments of social competence and interpersonal ease [70]. We note that all of the faces we used in our experiment displayed broad smiles and friendly expressions, which likely contributed not only to their perceived warmth but also their physical attractiveness [71]. We suggest that future studies investigate the effects of smiling vs. neutral scientist faces on perceptions of scientists’ warmth and competence, and the extent to which gender stereotypes might interact with those effects.

The positive effect of selfies on warmth stereotypes of scientists may also be dependent on viewers being motivated to individuate the scientists in these images and see them as human. We prompted such motivations by asking participants to evaluate individual scientist Instagrammers—their warmth, competence and attractiveness, how likely viewers would be to follow them in real life on Instagram, etc.—before asking participants to reflect on perceptions of scientists in general. When participants were asked questions about scientist stereotypes before evaluating individual Instagrammers, our stimulus had a much weaker effect on warmth stereotypes (see Table C in S1 Appendix). This is a limitation that should be investigated in future studies.

Another limitation of this study is that the results do not reveal whether or not scientists’ humanizing efforts on social media could have long-term impacts on stereotypes or even real-world impact on behavior. While one-time exposure to a series of humanized science images on IG reduced scientist and science gender stereotypes in our study, it remains to be seen whether this effect could extend beyond the experimental setting and persist over time. Likely important factors for this would be long-term or repeated exposure, two-way communication (e.g. scientist Instagrammers spending time replying to comments and engaging with followers), and meaningful relationship building between viewers and counterstereotypic scientists. These factors are theoretically supported by Instagram (through hashtag browsing, following, comments and replies, IGTV live broadcasts from the lab/field, etc.), but we did not incorporate these factors into this experimental design. Long-term, frequent, and meaningful exposure to counterstereotypic examples appear to be key to building trust and changing stereotypes long-term [62].

Juxtaposed to the concern about whether impacts of humanized science IG posts can be long-lasting is the concern that those spreading pseudoscience could leverage our “scientist selfie” approach to boost their own trustworthiness. There is no regulation as to who can use Instagram as a “scientist”; many have exploited scientific jargon and paraphernalia online to gain undue credibility and to spread pseudoscientific information. Some scientists have called for Twitter and Instagram to “verify” them (with a blue Verified badge) as it does other media figures and celebrities. However, a top-down approach to identifying “scientists” for verification could have the side effect of reinforcing traditional conceptions of who counts as a scientist. Considering that minorities in science may also be more likely to be boundary spanners, that women and minority scientists tend to be much less represented in the media, and that women scientist communicators accrue fewer social media followers [72], platform-initiated verification of scientists would be inherently problematic. We can instead support and amplify, as a community, grassroots humanized content from self-identified scientists on Instagram and other social networks to challenge science stereotypes, and train scientists in social media marketing to increase their reach. Self-identified scientists can use other source credibility cues (credentials, links to academic websites, references to peer-reviewed literature, etc.) to establish their credibility and raise awareness of how scientific credibility should be evaluated.

In summary, we believe that the power of selfies lies in humanizing and individuating scientists of all appearances who display warmth traits (smiling, eye contact, self-disclosure, sincerity, etc.). Any real-life scientist’s smiling face is likely to be perceived as warmer than common perceptions of scientists as peculiar, unfamiliar, and unfriendly. When it comes to smiling, however, we acknowledge gender bias that leads to women being asked to “smile” for the benefit of others, a request not often made of men. We do not endorse any use of our findings to bolster such bias. Finally, there are many ways for scientist communicators to display warmth, honesty, and shared values with audiences. The more diverse the approaches, the greater the potential reach. To connect on a human level through a smiling “selfie” is just one promising approach among many.

### Implications

By humanizing themselves on social media, scientists may be able to increase trust, public support, and interest in science. Scientists might help viewers individuate them by creating personally curated content with detailed captions, regularly engaging with audiences (asking questions, etc.), and sharing content that highlights “real life” as much as the scientific process. Selfies on Instagram posted by scientists from underrepresented groups may help others better “see” themselves in science and thereby help increase diversity and inclusion in STEM. Schinskie and colleagues [73] found that college students’ stereotypes of scientists are informed by their exposure to minority scientists in real life. During the course of this study, we heard from several educators who were using public #ScientistsWhoSelfie posts in their classrooms. For example, 8th graders at Woodstock Union Middle School in Woodstock, Vermont were challenged to reflect on their existing perceptions of scientists, through a Draw-A-Scientist test, and then explore photos of real scientists posted with the #ScientistsWhoSelfie hashtag on Instagram (R. Becker, personal communication). The students reflected on how their original drawings compared to the #ScientistsWhoSelfie photos. Many observed that scientists they saw in the photos, versus the ones they had drawn, did not fit a particular mold (e.g. white lab coats) but rather looked and dressed like “normal people” [74]. These anecdotal observations correspond with our own findings that selfies in scientific settings can help shape more positive perceptions of scientists. This is perhaps partly a result of exposure to a diverse set of faces that don’t fit traditional stereotypes.

This study is among the first to realistically address the usefulness of selfies for science communication. Previous research has explored various obstacles and training needs associated with scientists’ use of social media [75]. We suggest that scientists’ use of Instagram and selfies can positively impact how they are perceived. Our results add to a growing body of evidence that scientists can benefit from using social media. Scientists’ online presence can increase the visibility of their research [76], promote collaboration opportunities, improve research efficiency, enhance professional networking, and, in light of our findings, help promote public trust. STEM communities on social media might be able to meaningfully change science stereotypes by taking control of online representations of scientists, humanizing them and bolstering their online visibility. Institutional support for such efforts will be key to making what is good for public perceptions of scientists be good for scientists, too. This is especially true for women and minorities in STEM, who are often subjected to harsh online criticism and yet are pushed into public engagement responsibilities, without any formal reward or recognition. Where public engagement is forced, unwelcome or unrewarded, scientists may also have little motivation to communicate with warmth.

## Supporting information

S1 Dataset Full dataset for Qualtrics panel survey experiment. (ZIP)

S1 Appendix Supporting information for “Using selfies to challenge public stereotypes of scientists, including Tables A-D. (PDF)
