# Supplementary material for: Using selfies to challenge public stereotypes of scientists
Source: PLoS One. 2019 May 10;14(5):e0216625. doi: 10.1371/journal.pone.0216625 (PMC6510418; doi:10.1371/journal.pone.0216625)
Supplement: S1 Appendix — (PDF) [file pone.0216625.s002.pdf]

Supporting Information for

Using selfies to challenge public stereotypes of scientists

Paige Brown Jarreau[1,2], Imogene Cancellare[3], Becky Carmichael[2], Lancer Porter[4], Daniel Toker[5], Samantha Yammine[6]

Affiliations: [1]LifeOmic; [2]Louisiana State University, LSU College of Science, LSU CxC; [3]Department of Entomology and Wildlife Ecology University of Delaware; [4]Manship School of Mass Communication, LSU; [5]Helen Wills Neuroscience Institute, UC Berkeley, [6]Donnelly Centre for Cellular and Biomolecular Research, University of Toronto.

Corresponding Author: Paige Jarreau
Email: paigebjarreau@gmail.com

This PDF file includes:

Supplemental text
Tables A - D
References for S1 reference citations

Table of Contents

Supplemental Background ... *3*

Beyond the Deficit Model: When Perceptions of Scientists Matter ... 3

Visuals in Science Communication ... 3

Instagram ... 4

Supplemental Methods ... *6*

Stimulus Photos and Captions ... 6

Guidelines for Study Photos Contributions ... 7

Lab Pilot Study Supplemental Methods and Notes ... 11

Qualtrics Panel Supplemental Methods - Quotas ... 12

Qualtrics Panel Survey Instrument ... 13

Supplemental Results ... *18*

Pilot Study Results ... 18

Qualtrics Panel Study Supplemental Results ... 22

Supplemental Discussion ... *26*

Pilot Study Discussion ... 26

Qualtrics Study Discussion ... 26

Supplemental Tables ... *27*

Supplemental References ... *31*

Supplemental Background

Beyond the Deficit Model: When Perceptions of Scientists Matter

The scientific community has long focused on informational and consensus messaging (embodied by terms like "science says") and subscribed to the belief that increased scientific knowledge leads to more support for science, e.g. the "deficit model"[1]. However, the deficit model has come under considerable criticism as scientific knowledge has fallen short of explaining attitudes toward science[2]. We now understand that to know science is not necessarily to love it, and that many factors outside of knowledge influence people's perceptions of and attitudes toward science including cultural cognitions, identity, values and belief systems[3], gender[4], political ideology[5], media use[6], personal/educational experiences with science[7], and as we are studying here, perceptions of scientists. Stereotypes that scientists are competent but not warm can be significant communication barriers for scientists trying to engage public audiences[8]. We should address scientists' image problem if we are going to encourage scientists to communicate more often and directly with the public. Now more than ever we need to humanize scientists, diversify their public image and make their warmth traits more visible. Visual storytelling may be one way for scientists to help do this, especially if they can harness psychological processes known to foster individual/group warmth evaluations based on friendly faces and expressions.

Visuals in Science Communication

Visuals can either promote an oversimplification of science, as in popular depictions of DNA[9], or help promote an understanding of science, who scientists are and what they do on a daily basis. Researchers have advocated for the use of visual literacy paradigms in science communication since at least the late 1990s[9,10,11]. Science bloggers have advocated for matching the style of visual imagery to particular science communication goals, for example using cartoons to deflate anxieties that are scientifically unfounded (such as fear of vaccines or GMO-foods)[12]. But there has been very little

research on the types of visual communication in science that can accomplish the goals of building trust and changing stereotypes.

Instagram

Scientists and citizens alike are increasingly using social media to get and spread news and information about science[13], yet few peer-reviewed research studies have explored how social media and interactions between scientists and citizens in social media environments are shaping perceptions of scientists. We use Instagram, a photo and video sharing platform released in 2010, as an experimental platform to present and measure the impacts of friendly self-images of scientists on public science stereotypes. As McIntyre and colleagues have pointed out[14], modern social media networks are perfect venues to study the impact of the growing number of non face-to-face yet very visual and social interactions that occur between individuals of different groups, and how these drive social cognition. Traditionally, science museums and outreach events such as science cafes and fairs have been the only real opportunities for scientists and citizens to meet, exchange knowledge and begin to build mutual understanding and trust. However, Instagram is a space where scientists can connect on a personal level with non-experts. Instagram encourages public as well as one-on-one sharing of visual stories, particularly personal stories, and allows viewers to enter the Instagrammers' worlds and get to know them through selfies and self-videos.

The percentage of U.S. adult users using Instagram (18 to 50+ years of age) increased from 28% in 2016 to 35% 2018[15], with 60% of users visiting the platform daily. A whopping 71% of Americans between 18 to 24 years are Instagram users[15]. Instagram has fostered the development diverse communities around many topics and interests, including around science. A number of large scientific institutions and agencies regularly post to Instagram as a form of outreach, including NASA, with 33.9 million followers, the European Space Agency, CERN and the Society for Neuroscience. Scientists are also using Instagram, although in smaller in numbers than for Twitter. Celebrity scientists on Instagram include Neil Degrasse Tyson, Bill Nye, and Mitchell Moffit and Gregory Brown of YouTube's

AsapScience (@asapscience, 511k followers). Early scientist adopters have also gained large followings: Aaron Pomerantz (@nextgenscientist, 31.9k followers) and neuroscientist Samantha Yammine (@science.sam, 28.9k followers) are examples. With its one billion monthly active users as of June 2018 (https://instagram-press.com/our-story/), Instagram holds great potential for bolstering trust in scientists and scientific content if scientist Instagrammers can reach broad audiences with humanized visuals that promote perceptions of scientists' warmth, sociability, helpfulness and honesty. Instagram's culture of self-disclosure and humanization through "selfies" or self-portraits may further reinforce its potential as a tool for for science communication that builds public trust, by making the warmth traits of scientists more visible.

Both to promote this study and to increase friendly images of scientists on Instagram, we introduced the account @scientistselfies and the hashtag #scientistswhoselfie to social media in August, 2017. As of August, 2018, over 8k posts with the hashtag, and growing, have since been uploaded to Instagram by diverse scientists around the world.

Supplemental Methods

Stimulus Photos and Captions

All of our stimulus photos and captions were created by scientists whom we recruited via Instagram. In early 2017, we publicly invited scientists on Instagram to create a series of photos for our study based on a specific set of guidelines. See the next section for photo submission guidelines. We received submissions from more than 50 different scientists. We narrowed them down based on similarity between photos in a series. Volunteers (n = 15) helped us rate each photo series based on the quality of the photos, how interesting the photos were and how similar the photos were in composition, facial expressions/gestures, apparent age/ethnicity/physical attractiveness, scientific elements, etc. We used seven of the highest rating (in similarity and quality) images series in studies. These photo series varied in terms of field of science depicted (one physics, two cellular biology, two plant science/ecology, two animal/field biology) and scientist race/ethnicity in selfie photos (two hispanic, two middle eastern, two white/caucasian). Scientists were identified by name, age and location in IG post captions, e.g. “Deboki, 27 years old, Boston.” All scientists were depicted as being under 35 years of age.

For our pilot study, stimulus IG photos and captions were published to “Scientists of Instagram” IG accounts over time and then recycled throughout the data collection phase to keep the posts current. Participants viewed the posts in different orders depending on the date of data collection. Stimulus IG accounts all included the same avatar and bio, reading “a new #RotationCuration account featuring a different scientist every Monday and Friday!” For our online survey experiment, all of these IG posts were embedded into single scrollable web-pages for easy viewing, always in the same order (see scientistsofig.com - pages weren’t indexed on the homepage during the experiment). We did this to avoid issues of stimulus viewing via the IG platform for Qualtrics participant, such as the fact that our stimulus accounts were necessarily private.

Guidelines for Study Photos Contributions

We collected stimulus photos from scientists on Instagram, by promoting an opportunity to contribute to a study on perceptions of scientists. Participating scientists were instructed to create a photo of a scientific object or workstation in the lab or field, with a caption authentically and accurately describing the science/research they were working on with some accessible but specific details, and then recreate that photo with a) themselves in the shot; b) a stand-in colleague or friend of opposite sex in the shot. Participants were given the following instructions when they expressed interest in contributing to our project:

> Thank you for contributing content to our research project to study public perceptions of scientists on Instagram! Please submit at least 3 photos (1 set) and a scientific caption. Feel free to submit other 3-image sets if you have time! Deadline: July 1, 2017. E-mail raw high-resolution photos and captions to [redacted]. *NOTE: Do not post these photos on your own Instagram.
>
> **Procedures**:
>
> To contribute photos that will be included in this project, please follow the following procedures: 1. Take an interesting science-only photo. 2. Take the same photo, but include your smiling face, looking at the camera. 3. Take the same photo, but include the smiling face of someone who is of a different gender than you (who is representing you as the scientist in this photo), looking at the camera.
>
> **Steps**:
>
> Step #1. Take a photo of a visually interesting specimen, piece of equipment, workspace, science product/process, fieldwork scene, or something else you work with in a scientific lab or field environment. Step #2 requires you to take this same photo, but including your own face. You may need to prop up your photo to take this picture, such that you can easily insert yourself in the photo for Step #2 without changing the other elements of the photo. For example, if you take a

photo of a microscope with a sample under it, set up your phone/camera to the side of the mictroscope with enough space in the photo that you could easily insert yourself into the photo without changing the phone/camera angle or position drastically. (Example of a photo that could be reproduced easily with and without the human element: https://www.instagram.com/p/BQ0T7dPhap1/?taken-by=lsuscience)

Examples:

1a 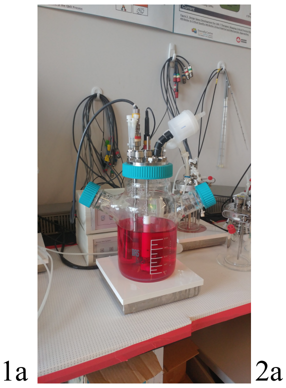 2a 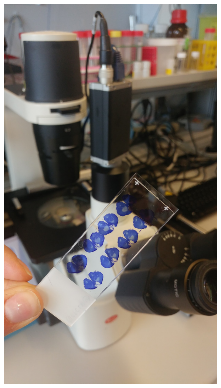

Step #2. Reproduce the photo you took in Step #1, but this time include yourself (your face) in the photo. The photo must include your smiling/happy face (We should see your eyes and smiling face, but if photo calls for looking somewhere not directly at the camera, e.g. at a specimen in your hand, that is OK) The main subject of your photo from Step #1 should still be clearly visible/distinguishable in the photo. Other elements should remain unaltered if possible. For example, if you took a photo of a petri dish on the lab bench, you might be holding this petri dish titled toward in the camera in this second photo, so that the same elements are still prominent. Try to keep the background and other elements in the photo as similar to the photo in Step #1 as possible. If possible, take this photo as both a normal photo (where your camera is propped up, on a tripod, or someone else is holding the camera) AND as a "selfie" (e.g. holding the camera). In this case, you'll produce two versions of this photo.

1b 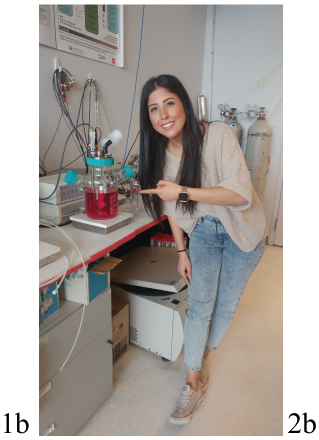 2b 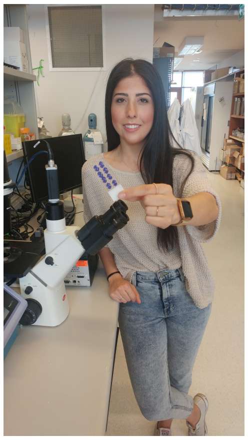

Step #3. Reproduce the photo you took in Step #2, but instead of having your face in the photo, include the face of a colleague or student around your age (and similar ethnicity) who is of opposite/different sex/gender than you. As much as possible, please have the person in this photo use the same stance, position, gestures and eye direction/contact as you did in Step #2. If possible, they should be wearing similar clothing or protective equipment as you did in Step #2. If possible, take this photo as both a normal photo (where your camera is propped up, on a tripod, or someone else is holding the camera) AND as a "selfie" (e.g. holding the camera). In this case, you'll produce two versions of this photo.

1c 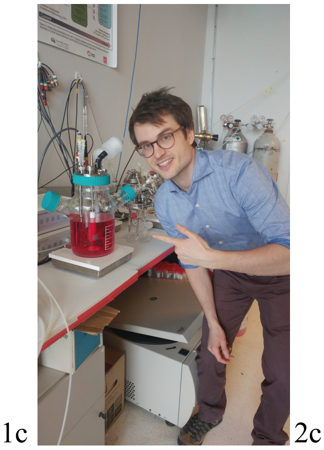 2c 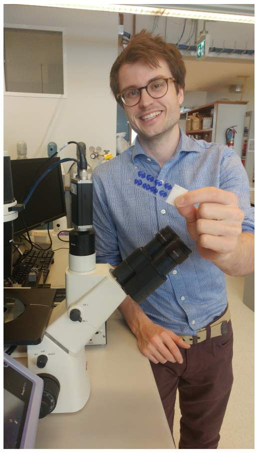

Note: Photos for Steps #2 and #3 should be selfie-like in nature, even if the person in the photo isn't technically holding the camera/phone. The person in the photo should be looking at the

camera and smiling. Clothing: Please wear field-appropriate clothing and all appropriate PPE (protective equipment like lab coats, gloves, etc. as appropriate for your field/work.) Please avoid accessories that dramatically stand out between the person included in Step #2 vs Step #3 photos (like jewelry, heavy make-up, other "gendered" accessories, etc. unless these are used in both photos.)

Step #4 - Caption. Include a descriptive, first-person scientific caption that can apply to all three photos. In the caption, include a lay summary of the science that is happening in the photo, relevant details of how you are conducting the science/research, and other context necessary to fully understanding what is happening in the photo. Also please include a sentence or two about the relevance, importance and/or broader implications of the research. This caption should be written such that it is understandable at a 6th-grade reading level.

Also include location data / country where you live or are doing science.

Photo guidelines:

- All photos should be original and created by you.
- Take photos in portrait mode / vertical (on your mobile device or camera). **Note - Instagram will slightly crop vertical photos from top and bottom, so try to leave "room" at top and bottom (or center the most important elements / faces in the photo).
- Do not apply any filters to the photos, or other editing techniques.
- Take photos you could find interesting, visually-appealing, or that might make you go "wow" if you saw them from another scientist on Instagram.
- Use lighting effectively. In most cases, that means using very well-lit conditions, in rooms or spaces with lots of ideally natural light. Other visually interesting lighting conditions specific to the conditions of your research might also be appropriate (for example, see the lighting here: https://www.instagram.com/p/BRbGfIFBmPT/?taken-by=lsuscience)

Lab Pilot Study Supplemental Methods and Notes

In a pilot study, students at a major state university viewed experimental science Instagram accounts designed to look like Rocur (rotation/curation) accounts featuring a different real-life scientist every week. We recorded a total of 162 research participant survey responses. A majority of the students were female (70%) and white/caucasian (83%), had taken fewer than three science courses in college (58%), and were Instagram users (89%).

Students came into a computer lab study space on campus where we (a female researcher and a student research associate, male in roughly half of the data collection sessions and female in the other half) handed them iPod Touch devices and gave them a minimum of eight minutes to browse content from either a control IG account or an experimental science IG account. Browsing of this content occurred via researcher-created, private “viewing” IG accounts, one for each iPod Touch. Each viewing account followed one of our private experimental science IG accounts, or a public control account, and was followed by six other accounts (our personal accounts) for external validity.

All students received equivalent bonus points in their respective courses for participating in the pilot study. For stimulus exposure, we had students use iPods to access private experimental Instagram accounts. iPods were in “guided access” mode to prevent navigation to the “explore” and “profile” tabs of Instagram, but otherwise students were allowed to browse our experimental IG content from the “viewing” accounts in any way they wished. Students were instructed to “like” and comment on posts freely. After at least eight minutes of browsing, we instructed students to complete a survey about their impressions of the Instagrammers they encountered during the exercise.

After running into an issue of students following hashtags in stimulus Instagram posts to other accounts and then following/subscribing to non-target accounts, the researchers began to instruct students not to follow or subscribe to any other Instagram accounts from their devices. We also occasionally ran into issues of students “liking” so many of the stimulus account posts that our user accounts were

temporarily blocked from “liking.” We navigated this issue by reporting the blocked action to Instagram after each data collection session and waiting 24-48 hours between data collection sessions.

Qualtrics Panel Supplemental Methods - Quotas

Survey respondents were recruited and screened for meeting participant quotas by Qualtrics. Our goal was 1,500 survey responses, or 300 per experimental condition plus a control condition, with U.S. representative quota constraints for gender (51.79% female), age (12.06% 18-24 years, 17.69% 25-34 years, 17.06% 35-44 years, 18.29% 45-54 years, 16.53% 55-64 years, 18.37% 65+ years), and education (13.32% less than HS diploma, 28.02% HS diploma/GED, 23.14% some college (no degree), 7.81% associate's degree, 17.63% bachelor's degree, 10.08% graduate degree).

Qualtrics Panel Survey Instrument

<u>Pre-stimulus / Screener Questions</u>

❏ Sex: What is your biological sex [male/female]

❏ Age: How old are you? [age in number]

❏ Education: What is the highest degree or level of school you have completed?
- Completed some high school or less
- High school graduate
- Completed some college
- Associate degree (AA, AS)
- Bachelor's degree (BA, AB, BS)
- Completed some postgraduate
- Master's degree (MA, MS, MEng, MBA)
- Professional degree (MD, DDS, DVM, LLB, JD)
- Doctorate degree (PhD, EdD, etc.)

❏ Degree field [dropdown]
- Agriculture, Forestry, Horticulture, Environmental sciences
- Business, Finance, Marketing, Accounting, Economics or related field
- Computer/Information science
- Education
- Engineering
- Law
- Liberal Arts
- Life science, Health science or Medicine
- Mass Communication - Journalism
- Mass Communication - Public Relations, Advertising, Strategic communication, etc.
- Mathematics/Statistics
- Physical science - Astronomy, Atmospheric science, Chemistry, Earth science, Physics, etc.
- Psychology/Behavioral science
- Other Social Science
- Other

❏ Topic Interest: How interested are you in information about each of the following topics? *Please answer on a scale of 1 (Not at all) to 5 (Extremely).* [order randomized]
- Government and politics
- Sports
- Science (biology, physics, etc.)
- Business and finance
- Health and medicine

Stimulus Exposure

Conditions: (1) science only, by males, (2) science only, be females, (3) science + male face, (4) science + female face, (5) control (posts from Humans of Broadway Instagram account).

- ❑ READ CAREFULLY: To fully participate in this study, you must look and read through a series of Instagram posts representing different groups in society, and answer questions about your perceptions of the individuals and information you encounter.

  The URL below will open in a new window a webpage containing screenshots of Instagram content. Once you are finished looking and reading through the Instagram posts, return to this window and continue for a questionnaire about the Instagram content you saw. You will be asked what you remember most from the Instagram posts.

  Click URL >> ScientistsofIG Instagram Posts

  *You may need to wait a few moments for the photos on the webpage to load.

- ❑ Stimulus viewing confirmation: Did you click the link above to view the Instagram content? If so, tell us briefly but descriptively what you saw and read (you must enter a meaningful text response based on what you remember to continue the survey). [“Yes” response and open-ended entry required to continue survey.]
  - Yes and I saw___ (open-ended)
  - No

Post-Stimulus Online Questionnaire

- ❑ Stimulus Content Enjoyment: *Please indicate your agreement with the following statements, on a 5-point scale from 1 "strongly disagree" to 5 "strongly agree.”* [order randomized]
  1. I would describe the Instagram posts as very interesting.
  2. I enjoyed looking at the Instagram posts.
  3. I enjoyed reading the captions of the Instagram posts.
  4. I thought the Instagram posts were boring.
  5. The Instagram posts sparked my curiosity.
  6. The Instagram posts provided credible information.
  7. The Instagram posts provided useful information.
  8. I learned a lot from the Instagram posts.
  9. I want to see more posts from the Instagram account.

Perceptions the Instagrammers: The following questions relate to the individuals or Instagrammers that you encountered through the posts you looked and read through, whether they took the photos or were shown in the photos.

- ❑ Value similarity (via Siegrist et al., 2000): *Please indicate in the following pairs of opposite words how similar or dissimilar you think you are compared to the Instagrammers you encountered through this activity*. [order randomized]
    1. Different values – Same values
    2. Different goals – Sam goals
    3. Thinks unlike me – Think like me
    4. Different opinions – Same opinions
    5. Different beliefs – Same beliefs

- ❑ Instagrammer Trustworthiness: *Please indicate your agreement with the following statement: I believe the individuals/Instagrammers I encountered through the Instagram posts are very trustworthy.* (1 = strongly disagree, 7 = strongly agree)

- ❑ Look Like Scientists: *To what extent do you think the individuals/Instagrammers you encountered looked like scientists?* (1 = not at all, 7 = a great deal)

Screenshot Evaluations of Individual Instagrammers (4 images and corresponding questions presented one-by-one): We are now showing you select photos from the Instagram posts that you looked and read through at the beginning of this survey. Please answer the questions provided immediately after you see each image by thinking about what you saw in the image.

Example:
Seaweed science, with either a science-only image if participants had browsed science-only stimulus IG posts, a male selfie is participants had browsed male selfies during stimulus exposure, a female selfie if participants had browsed female selfies during stimulus exposure.

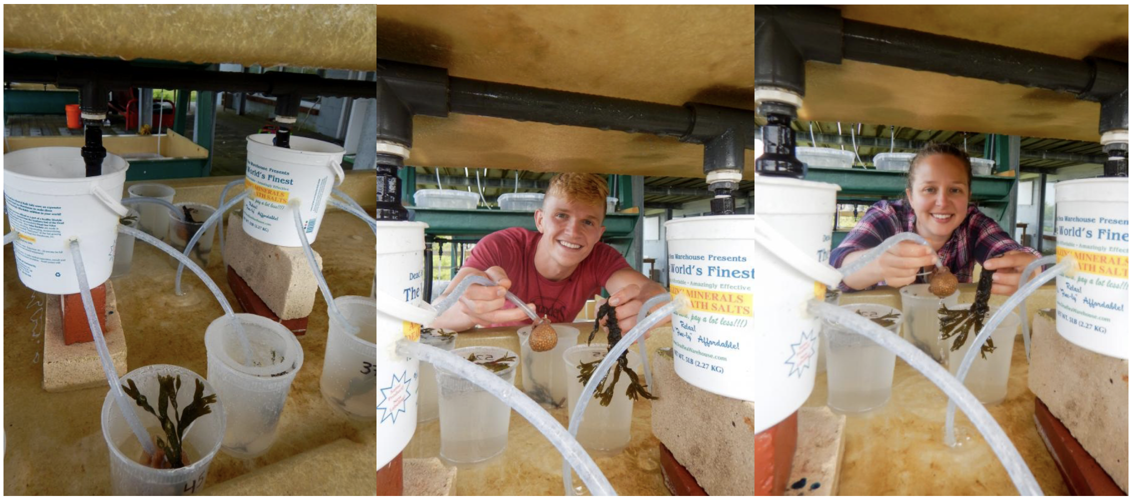

- ❑ Warmth: *Please respond to the following statement on a scale of 1 (not at all) to 5 (extremely)*: The person in this (selfies) / who took this (science-only) photo is warm.

- ❑ Competence: *Please respond to the following statement on a scale of 1 (not at all) to 5 (extremely)*: The person in this (selfies) / who took this photo (science-only) is competent.

- ❑ Attractiveness: How physically attractive do you think the person in this photo is? (selfies) OR If you had to guess, how physically attractive do you think the person who took this photo is? (science-only). (1 = very unattractive, 3 = Neutral, 5 = very attractive)

- ❑ Follow: How likely would you be to follow this individual on Instagram in real life (if you have or if you had an Instagram account)? (1 = extremely unlikely, 5 = extremely likely).

Perceptions of Scientists: In this section, we are interested in learning how different groups of people are viewed by society.

- ❑ Warmth vs Competence (via Fiske, Cuddy & Glick, 2002; Fiske & Dupree, 2014): *Please respond to the statement below by indicating, on a scale of 1 (not at all) to 5 (extremely), how well each of the following words represent traits of* ***scientists****, in general. Generally speaking, scientists are…*

- Narcissism related terms: Self-focused, Vain, Modest
- Warmth related terms: Altruistic, Sincere, Honest, Dishonest, Warm, Helpful, Sociable, Ethical, Unethical, Likeable, Friendly, Trustworthy
- Competence related terms: Competent, Confident, Competitive, Intelligent
- Physically attractive

- ❑ Symbolic threat: If resources go to scientists, to what extent does that take resources away from the rest of society? (1 = Not at all, 5 = A great deal).

- ❑ Gender stereotypes: *Please rate how much you associate the following domains with males or females:*
  Liberal Arts [strongly male, somewhat male, neither male nor female, somewhat female, strongly female]
  Social media use [strongly male, somewhat male, neither male nor female, somewhat female, strongly female]
  Science [strongly male, somewhat male, neither male nor female, somewhat female, strongly female]

- ❑ Know a scientist: Do you know a scientist personally? [Yes, No]
  - ❑ If YES: Please name any scientists whom you know personally [open-ended]

Science News and Demographics.

- ❑ Science News: How often do actively seek out science-related content or information online? (1 = Never, 5 = Very frequently).

- ❑ Science Sources: Which of the following, if any, do you regularly get science-related content, information or news from? [Select all that apply]

- News outlets that cover a range of topics
- TV
- Science magazines
- Science podcasts or radio programs
- Science and technology centers or museum
- Social networking sites (e.g. Facebook, Twitter, Instagram)
- Other websites and blogs focused on science
- None of these

- ❏ Social Media: Do you ever use social media (such as Instagram, Snapchat, Twitter, Facebook, etc.)? [Yes, No]

- ❏ Instagram: Do you have an Instagram account? [Yes, No, I don't know]

- ❏ Frequency of IG Use: How often do you use Instagram in any capacity (posting or browsing)? (1 = never, 5 = 4-6 times a week)

- ❏ Frequency of Social Media Use: How often do you use social media? (1 = less often / never, 5 = several times per day)

- ❏ Follow Scientists: On social media, do you follow any people that are focused on science, such as scientists? [Yes, at least one, No]

- ❏ Science Social Media: How often do you see science-related information or content on social media (through Facebook, Twitter, Snapchat, or Instagram, or on blogs, Reddit, etc.)? (1 = Never, 5 = Very frequently)

- ❏ Gender: I identify as… [Male, Female, Other, Prefer not to answer]

- ❏ Ethnicity: I would describe myself as… [American Indian/Native American, Asian, Black/African American, Hispanic/Latino, White/Caucasian, Pacific Islander, Middle Eastern, Other, Prefer not to answer]

- ❏ Political affiliation: I usually think of myself as a… [Republican, Democrat, Independent, Something else [text]]

- ❏ Religious affiliation: *How important is religion in your life?* [very important, somewhat important, not too important or not at all important?]

- ❏ Science education (# of science classes taken in college): Approximately how many science classes (or classes in science, technology, engineering or math disciplines) have you taken in college? [0-10+]

Supplemental Results

Pilot Study Results

In a pilot study, students at a large university browsed control IG posts (via the public "Humans of Southeastern" account) and experimental posts (four conditions: science-only male, science-only female, selfie male, selfie female) on iPod Touch devices via private IG viewing accounts created for the purpose of the experiment (between-subjects experimental design). See supplemental methods section for more details. The final analysis was based on a sample of 162 undergraduate students, most of whom were majoring in mass communication or business disciplines (62%).

Enjoyment. There was a main effect of stimulus condition on enjoyment (Control vs Science-only vs Selfie posts). Participants indicated significantly more enjoyment of content from control IG accounts that from experimental science IG accounts (Analysis of variance, $F(2,159) = 7.82$, $p < .001$). In a post-hoc analysis with Bonferonni adjustment for multiple comparisons, enjoyment scores for control IG posts were significantly higher than enjoyment scores for Science-only or Selfie IG posts (Control vs Science-only mean difference = .59, $p < .01$; Control vs Selfie mean difference = .66, $p < .01$). Enjoyment scores for Science-only and Selfie IG posts were comparable (Science vs Selfie mean difference = -.07, $p = 1.00$). Enjoyment means and standard deviations: Control = 3.58(.80); Science-only = 2.98(.84); Selfie = 2.91(.81)).

Information Quality. There was a main effect of stimulus condition on information quality, consisting of perceptions that the content was credible, useful, educational and valuable (Control vs Science-only vs Selfie posts). In an analysis of variance (*Welch* $F(2, 85.66) = 27.74$, $p < .001$) students perceived information provided in both science-only ($M = 3.75$, $SD = .60$, $n = 66$, $p < .001$) and selfie IG posts ($M = 3.76$, $SD = .59$, $n = 63$, $p < .001$) to be significantly more credible, useful, educational and valuable (4-item scale, Chronbach's alpha = .79) than information provided in control IG posts ($M = 2.89$, $SD = .59$, $n = 33$). In a post-hoc analysis with Bonferonni adjustment for multiple comparisons, Science-

only and Selfie IG posts were scored higher than Control IG posts (Science-only vs Control mean difference = .86, $p < .001$; Selfie vs Control mean diff = .86, $p < .001$), while Science-only and Selfie posts were scored comparably (Selfie vs Science-only mean difference = .01, $p = 1.00$). Information quality means and standard deviations: Control = 2.89(.59); Science-only = 3.75(.60); Selfie = 3.76(.59).

Perceptions of Instagrammers. Following stimulus browsing on iPod devices, we asked students to answer a series of questions about the content and the Instagrammers they had encountered. With screenshots embedded in an online questionnaire, we re-displayed select photographs from the experimental IG accounts students had browsed during stimulus exposure (visuals only, no captions) and asked them to rate the warmth and competence (single items) of the Instagrammers who had taken or who were in these photos[1], an individuating exercise. In a series of analysis of variance tests, there was a main effect of stimulus condition (science-only vs selfies) on perceived warmth (*Welch* $F(1,125.50) = 61.75$, $p < .001$) but not on competence (*Welch* $F(1,125.50) = .03$, $p = .85$). Competence (single item) means and standard deviations: Science-only = 3.88(.75); Selfies = 3.91(.76).

Students found Instagrammers in selfies to be significantly warmer (M(warm) = 3.74, SD = .71, n = 62) than Instagrammers posting science-only photos (M(warm) = 2.74, SD = .71, n = 66), $p < .001$. There were no significant differences in competence. In an analysis of variance including perceived physical attractiveness of the Instagrammer as a covariate, students found female scientists in selfie photos to be slightly warmer (estimated mean = 3.71, SE = .13) than male scientists (estimated mean = 3.66, SE = .13), but the difference was not significant.

We also ran an exploratory linear regression to investigate other predictors of perceived warmth of individual scientist Instagrammers (mean perceived warmth of four Instagrammers who either took or were in stimulus photos; model summary: $R^2 = .42$; $F(8, 117) = 10.63$, $p < .001$). See results in Table A.

[1] In our pilot study, participants in experimental conditions were always asked to rate the warmth and competence of select Instagrammers they had encountered during stimulus browsing before they were asked to indicate their perceptions of scientists in general.

Significant predictors included stimulus condition (science-only versus selfie photos), enjoyment of the content and knowing a scientist personally. Students who had browsed science selfie IG posts, students who knew a scientist personally and students who expressed greater enjoyment of the experimental content rated the scientist Instagrammers as significantly warmer. In a similar regression model predicting perceived competence of scientist Instagrammers (model summary: $R^2 = .16$; $F(8, 117) = 2.73$, $p < .01$), only knowing a scientist personally and perceived attractiveness were significant predictors of competence. Stimulus condition was not a significant predictor.

Fields of Science. Of interest, participants evaluated most individual Instagrammers who had taken Science-only images to be only moderately warm at best (consistently between a score of 2.7 and 3 on a 5-point scale), while evaluating them as highly competent (around a score of 4 on a 5-point scale). However, some of the lowest warmth scores (Male Science = 2.30(.81); Female Science = 2.44(.88)) were attributed to scientists posting photos of cell counts / flow cytometry graphical results on a computer screen. It could be that scientists communicating about and posting more relatable science and/or less technical scientific images (photos of colorful items on a lab bench, of an experimental set-up with plants, of coral reefs, etc.) are naturally perceived as warmer than scientists communicating about and posting more technical images (graphs, scientific visualizations, etc.), although this concept needs further investigation.

Follow Intention. There was no significant effect of stimulus condition on intention to follow the encountered Instagrammers in the future (Analysis of variance, $F(2,158) = .933$, $p = .40$).

Evaluations of Scientist Instagrammers and Ethnicity. There was no obvious impact of ethnicity on perceived warmth of the scientist instagrammers. The two highest warmth scores belonged to caucasian male scientist and a hispanic female scientist. The caucasian male scientist (in the seaweed experiment IG post) had a particularly young-looking face, which could have contributed this scientist to receiving the high warmth ratings. The female hispanic scientist (coral reef IG post) also received a very high competence rating.

Male Selfie warmth means and standard deviations: Bioreactor (caucasian) = 3.55(.85); Seaweed experiment (Caucasian) = 4.29(.82); Coral reef (hispanic) = 3.16(.97); Cell biology (Indian) = 3.71(.82)

Female Selfie warmth means and standard deviations: Bioreactor (caucasian) = 3.84(.93); Seaweed experiment (Caucasian) = 3.84(1.0); Coral reef (hispanic) = 3.94(.89); Cell biology (Indian) = 3.57(.86);

Male Selfie competence means and standard deviations: Bioreactor (caucasian) = 4.00(.82); Seaweed experiment (Caucasian) = 3.97(.91); Coral reef (hispanic) = 3.77(.92); Cell biology (Indian) = 4.00(.78); .

Female Selfie competence means and standard deviations: Bioreactor (caucasian) = 3.68(.95); Seaweed experiment (Caucasian) = 3.81(.95); Coral reef (hispanic) = 4.00(.89); Cell biology (Indian) = 4.03(.91).

Evaluations of Scientist Instagrammers and Attractiveness. The scientist instagrammers were mostly well-matched by gender in terms of perceived attractiveness.

Value Similarity. There was no significant direct impact of stimulus condition on perceived value similarity between participants and the Instagrammers they had encountered (Analysis of variance, $F(2,157) = .184$, p = .162).

Stereotypes of Scientists. We also observed changes in scientist stereotypes with experimental stimulus exposure, particularly for selfies. There was a significant main effect of stimulus condition (Control vs Science-only vs Selfie posts) on stereotypes of scientists' warmth (measured as a single single item; no significant results of a warmth scale) (Analysis of variance *Welch* $F(2, 80.23) = 6.12$, $p < .01$; Levene's statistic = 3.26, $p < .05$), but not their competence (2-item scale combining perceived competence and intelligence; Analysis of variance *Welch* $F(2, 81.78) = 1.68$, p = .19).

In a post-hoc analysis with Bonferonni adjustment for multiple comparisons, students who had browsed selfie posts (M = 2.98, SD = .73, n = 63) also rated scientists in general as warmer (single item) than did students who had browsed science-only (M = 2.62, SD = .65, n = 66, Mean difference = .36, $p < .05$) or control IG posts (M = 2.48, SD = .83, n = 33, Mean difference = .50, $p < .01$). There were no

significance differences between these groups in terms of perceived competence, although students who had browsed either science-only or science selfie IG posts tended to rate scientists in general as more competent (Science-only M = 4.47, SD = .61; Selfie M = 4.5, SD = .57); than did students who had browsed control IG posts. Also, science-only posts did not result in greater perceived warmth of scientists as compared to Control posts (Science-only vs Control mean difference = .14, p = 1.00). Warmth (single item) means and standard deviations: Control = 2.48(.83); Science-only = 2.62(.65); Selfie = 2.98(.73). Competence (2-item scale) means and standard deviations: Control = 4.27(.61); Science-only = 4.48(.51); Selfie = 4.50(.60).

Scientist Gender Effects. There was no main effect of of scientist gender on stereotypes of scientists. There was a marginally significant main effect of scientist gender (female vs male scientists in experimental science IG posts) on science stereotypes. Students who had browsed IG posts depicting female scientists (science-only and selfies) had some positive but only marginally significant changes in their explicit gender science stereotypes; M(female IG) = 2.38, M(male IG) = 2.15, $p = .06$.

Qualtrics Panel Study Supplemental Results

Evaluations of Scientist Instagrammers and Attractiveness. Scientists in female selfies were consistently evaluated as significantly more attractive than scientists in male selfies.

Male Selfie attractiveness means and standard deviations: Bioreactor (caucasian) = 3.19(.93); Seaweed experiment (Caucasian) = 3.25(.88); Coral reef (hispanic) = 2.76(.87); Cell biology (Indian) = 2.90(.93).

Female Selfie attractiveness means, standard deviations and ANOVA statistics for comparisons of means to male Selfies: Bioreactor (caucasian) = 3.83(.95), $F = 76.12$, $p < .001$; Seaweed experiment (Caucasian) = 3.45(.85), $F = 8.61$, $p < .01$; Coral reef (hispanic) = 3.15(.89), $F = 30.92$, $p < .001$; Cell biology (Indian) = 3.22(.97), $F = 77.85$, $p < .001$.

Attractiveness of Female Instagrammers. We ran an ANCOVA analysis exploring attractiveness evaluations by stimulus scientist gender in stimulus IG posts. We included participant age

and sex as covariates. We found a significant effect of stimulus and scientist gender on attractiveness, such that scientists in selfies were evaluated as more attractive than scientists posting science-only IG posts (Stimulus $F(1, 1264) = 46.44$, $p < .001$), and female scientist IGers were evaluated as significantly more attractive than male scientist IGers (Scientist Gender $F(1, 1264) = 74.82$, $p < .001$). The effect of age was also significant ($F(1, 1257) = 23.56$, $p < .001$), while participant sex was not. Model $F(4, 1264) = 36.30$, $p < .001$).

Vanity of Scientists. We measured perceived vanity (single item, 5-point scale) of scientists, and found that only exposure to female selfies produced significantly lower perceptions of scientists as vain as compared to control images or science-only images (Anova F(4,1613)= 5.58, p < .001; Control M = 2.57, SD = 1.12, Sci Male M = 2.51, SD = 1.03, Sci Female M = 2.45, SD = 1.10, Selfie Male M = 2.36, SD = 1.10, Selfie Female M = 2.19, SD = 1.11).

Competence Ratings of Scientists, by Stimulus Condition and Participant Gender. We grouped ANCOVA analyses of stimulus impact on perceived competence of Instagrammers by participant gender. Average perceived attractiveness of the IGers was added as a covariate to these ANCOVA analyses. We found that male viewers (Stimulus $F(3, 553) = 2.45$, p = .06) perceived female scientists in Selfies as least competent, and less competent than male scientists posting Science-only photos to a marginally significant level ($p = .05$). Estimated means and standard errors among male participants: Science Male = 3.65(.06); Science Female = 3.57(.06); Selfie Male = 3.54(.06); Selfie Female = 3.40(.06). This trend was not as prominent among female viewers (Stimulus $F(3, 673) = 1.35$, p = .26). Estimated means and standard errors among female participants: Science Male = 3.66(.06); Science Female = 3.53(.06); Selfie Male = 3.50(.06); Selfie Female = 3.53(.06).

Warmth Ratings of Scientists, by Stimulus Condition and Participant Gender. We grouped ANCOVA analyses of stimulus impact on perceived warmth of Instagrammers by participant gender. We found that among male viewers (Stimulus $F(4, 701) = 3.01$, $p < .05$), only female selfies, not male selfies, resulted in significantly higher perceptions of scientists' warmth as compared to control images and science-only male images ($p < .05$). Estimated means and standard errors among male participants:

Control = 3.32(.05); Science Male = 3.29(.06); Science Female = 3.35(.06); Selfie Male = 3.36(.05); Selfie Female = 3.54(.06). Among female viewers (Stimulus $F(4, 858) = 3.64$, $p < .01$) a similar trend was observed, although male selfies resulted in slightly higher perceptions of scientists' warmth among female viewers than they did among male viewers. Female selfies seemed to have a similar effect on stereotypes of scientists' warmth for both male and female viewers. Estimated means and standard errors among female participants: Control = 3.31(.05); Science Male = 3.29(.05); Science Female = 3.32(.05); Selfie Male = 3.44(.05); Selfie Female = 3.50(.06).

Looks Like a Scientist. Both male and female scientist Instagrammers were evaluated similarly in terms of looking like scientists (a score of 3.30 out of 5 in both cases). These moderate scores may have been due to the individuals in the Instagram posts being relatively young and counterstereotypical of scientists in general (smiling, sociable) as compared to what people generally assume or have seen from media representations of what scientists look like.

Impact of Display Order on Scientist Warmth Stereotypes. To help elucidate mechanisms of the impact of selfies on stereotypes of scientists' warmth, we investigated warmth stereotypes by question order. Half of our participants (random assortment) viewed stimulus content and answered survey questions in the following order: Stimulus viewing at Scientistsofig.com > Evaluations of the individual Instagrammers via re-displayed stimulus images embedded in an online survey > Scientist Stereotypes. The other half viewed stimulus content and answered survey questions in the following order: Stimulus viewing at Scientistsofig.com > Scientist Stereotypes > Evaluations of the individual Instagrammers via re-displayed stimulus images embedded in an online survey.

When participants evaluated individual encountered Instagrammers first before answering questions about perceptions/stereotypes of scientists, there was a significant main effect of stimulus condition (Science-only Male vs Science-only Female vs Selfies Male vs Selfies Female) on warmth (ANCOVA, Stimulus $F(3, 634) = 7.08$, $p < .001$; Model $F(11, 634) = 9.30$, $p < .001$). When participants evaluated individual encountered Instagrammers after answering questions about perceptions/stereotypes of scientists, there was no main effect of stimulus condition on warmth (ANCOVA, Stimulus $F(3, 592) =$

1.89, $p = .13$; Model $F(10, 592) = 2.51$, $p < .01$). See Table C for post-hoc comparisons by stimulus condition.

Explorations of the Role of Individual Instagrammer Evaluations on Warmth Stereotypes. In linear regression analyses including a selfie vs. science-only stimulus variable and other factors as listed in Table B (Model 1) in the main manuscript, our stimulus variable had a non-significant impact on stereotypes of scientists' warmth (Stimulus $\beta = .04$, $p = .12$) when adding perceived attractiveness, and even a negative impact (Stimulus $\beta = -.10$, $p < .001$) when adding individual warmth evaluations. This is not surprising given that we believe the individuating exercise of evaluating individual scientist Instagrammers contributed to the stereotype changes we observed. We explored this in our manuscript through a mediation analysis (see Figure 2 in the main manuscript). Perceived attractiveness ($\beta = .35$, $p < .001$) and perceived warmth of scientist Instagrammers ($\beta = .59$, $p < .001$) are both strong predictors of scientist warmth stereotypes, and they are strongly correlated (Pearson coefficient = .46, $p < .001$). Factoring in enjoyment of the Instagram content, however, rescues significance of our stimulus variable when perceived attractiveness is in the linear regression model (Stimulus $\beta = .06$, $p < .05$). Both positive evaluation of individual scientists and enjoyment of their content appear to be important to positive stereotypes of scientists as a result of exposure to scientist selfies on Instagram.

Supplemental Discussion

Pilot Study Discussion

Limitations. In debriefing following our pilot study data collection sessions, we found that many students who participated in our pilot study did not realize during stimulus browsing that the primary content they were viewing consisted of posts *about scientists*. Many students ended up browsing, via hashtags in our stimulus posts, to off-target IG accounts. While this was a good sign for the external validity of our pilot study, it may explain why our content failed to broadly change students' overall stereotypes of scientists. It could also be that our science-related Instagram posts were not interesting enough to captivate viewers not particularly interested in science. In our pilot study survey instrument, we did not measure attention to or recall of IG post caption information, or whether participants perceived the Instagrammers to look like or actually be scientists. Future research should evaluate how real-life browsing of Instagram science influences perceptions of science and scientists, and what characteristics of Instagram posts might prompt viewers to spend more time viewing/reading captions and motivate them to follow and get to know scientist Instagrammers better.

Qualtrics Study Discussion

Role of Political Affiliation. We observed through linear regression analyses predicting scientist stereotypes that democrats perceive scientists as warmer than do republicans. The polarization of our current media environment (Stroud, 2011) could help explain partisan divisions in public opinions and trust in scientists. (Hmielowski et al., 2013)

Supplemental Tables

Table A. Pilot study results of linear regression analysis predicting scientist Instagrammer warmth and competence

Model	Warmth		Competence	
	β	95% CI of *B*	β	95% CI of *B*
Constant		[-.16, 2.35]		[-.91, 3.20]
Stimulus	.53***	[.65, 1.18]	-.06	[-.37, .19]
Scientist gender	.05	[-.15, .34]	-.10	[-.41, .11]
Enjoyment	.16*	[.003, .353]	.16	[-.03, .34]
Attractiveness	.13	[-.06, .48]	.22*	[.02, .59]
Participant gender	.11	[-.08, .49]	.13	[-.08, .53]
Participant age	.09	[-.04, .14]	.12	[-.03, .15]
Know a scientist	.16*	[.03, .53]	.24**	[.10, .63]
# of science classes	-.11	[-.08, .02]	-.09	[-.08, .03]
F total				
R^2				

Notes: β = standardized coefficient. B = unstandardized regression coefficient. CI = confidence interval. Only weak correlations are found between predictors: Instagrammer attractiveness and selfie stimulus condition are weakly correlated (Pearson coefficient = .32, $p < .001$), as are age and number of science classes completed (Pearson coefficient = .38, $p < .001$). Gender variables are coded as Male (0) vs Female (1).

*p < .05. **p < .01. ***p < .001.

Table B. Demographics of Qualtrics panel survey experiment respondents

Variable	n	Percent (%)	Valid Percent (%)
Race			
White/Caucasian	1227	75.8	76.5
Black/African American	145	9.0	9.0
Hispanic/Latino	97	6.0	6.1
Asian	56	3.5	3.5
American Indian/Native	24	1.5	1.5
Other	36	2.0	2.0
Prefer not to answer	18	1.1	1.1
Education			
Less than HS	171	10.6	10.6
HS graduate	427	26.4	26.5
Some college	524	32.4	32.5
Bachelor's	305	18.9	18.9
Some postgrad/ Master's	144	8.9	8.9
PhD / MD	42	2.6	2.6
Political Affiliation			
Republican	468	28.9	29.2
Democrat	519	32.1	32.4
Independent	497	30.7	31.0
Other	119	7.4	7.4
STEM degree	155	9.6	9.6
Gender			
Female	891	44.7	44.8
Male	724	55.1	55.2

Table C. Impacts of stimulus on scientists' warmth Stereotypes, by question order – ANCOVA analyses with post hoc estimated mean contrasts by stimulus group.

	Estimated marginal mean (standard error)					
	Science Male	Science Female	Selfie Male	Selfie Female	*F*	η2
Screenshots first	$3.25(.05)_{a}$	$3.22(.05)_{ab}$	$3.48(.05)_{bc}$	$3.55(.05)_{c}$	7.08***	.032
Stereotypes first	$3.35(.05)_{a}$	$3.34(.05)_{a}$	$3.32(.05)_{a}$	$3.48(.05)_{a}$	1.89	.010

Notes: Results based on SPSS GLM ANCOVA analyses. Covariates in models include participant gender, age, education, science interest, Instagram use, importance of religion, and knowing a scientist personally. Means with differing subscripts within rows are significantly different at the p < .05 based on Bonferroni post hoc pairwise comparisons.

*p < .05. **p < .01. ***p < .001.

Table D. Linear regression analysis predicting Instagrammer trustworthiness

Model 1	Trustworthiness	
	β	95% CI of *B*
Constant		[3.95, 4.69]
Selfie vs Control	.13***	[.22, .53]
Selfie vs Science	.12***	[.17, .43]
Participant gender	-.01	[-.12, .11]
Participant age	.05*	[.00, .01]
Participant education	.02	[-.03, .09]
Interest in science	.22***	[.17, .27]
Religion	.04	[-.01, .08]
Know a scientist	-.03	[-.26, .05]
Instagram use	.09**	[.02, .09]
Democrat vs Other	.12***	[.15, .45]
Indep. vs Other	.01	[-.14, .15]
Scientist Gender (Model 2)	.06*	[.02, .27]
F total	15.26***	
R^2	.10	

Notes: β = standardized coefficient. B = unstandardized regression coefficient. CI = confidence interval. Degrees of freedom for Model 1 regression equation are $F(11, 1569)$. Stimulus variable represents science-only posts versus selfie posts. Only weak correlations are found between predictors: Instagram use and age (Pearson coefficient = -.36, p < .01). Dummy variables are coded as X (1) vs Other (0). Gender variables are coded as Male (0) vs Female (1).

*p < .05. **p < .01. ***p < .001.

Supplemental References

1. Bauer MW, Allum N, & Miller S (2007) What can we learn from 25 years of PUS survey research? Liberating and expanding the agenda. *Public Understanding of Science* 16(1):79-95.
2. Sturgis P & Allum N (2004) Science in Society: Re-Evaluating the Deficit Model of Public Attitudes. *Public Understanding of Science* 13(1):55-74.
3. Kahan DM, Jenkins SH, & Braman D (2011) Cultural cognition of scientific consensus. *Journal of Risk Research* 14(2):147-174.
4. Osborne J, Simon S, & Collins S (2003) Attitudes towards science: A review of the literature and its implications. *International Journal of Science Education* 25(9):1049-1079.
5. McCright AM, Dentzman K, Charters M, & Dietz T (2013) The influence of political ideology on trust in science. *Environmental Research Letters* 8(4):044029.
6. Ho SS, Brossard D, & Scheufele DA (2008) Effects of Value Predispositions, Mass Media Use, and Knowledge on Public Attitudes Toward Embryonic Stem Cell Research. International Journal of Public Opinion Research 20(2):171-192.
7. Kind P, Jones K, & Barmby P (2007) Developing Attitudes towards Science Measures. *International Journal of Science Education* 29(7):871-893.
8. Fiske ST & Dupree C (2014) Gaining trust as well as respect in communicating to motivated audiences about science topics. *Proc Natl Acad Sci USA* 111(Supplement 4):13593-13597.
9. Trumbo J (2000) Essay: Seeing Science:Research Opportunities in the Visual Communication of Science. *Science Communication* 21(4):379-391.
10. Trumbo J (1999) Visual Literacy and Science Communication. *Science Communication* 20(4):409-425.
11. Estrada FCR & Davis LS (2015) Improving Visual Communication of Science Through the Incorporation of Graphic Design Theories and Practices Into Science Communication. *Science Communication* 37(1):140-148.

12. Mellow, Glendon (2016). Using Science Art and Imagery in a Blog. Science Blogging: The Essential Guide. Eds. Christie Wilcox, Bethany Brookshire, & Jason G. Goldman. (Yale University Press).
13. Pew (2017) Science News and Information Today. (Pew Research Center).
14. McIntyre K, Paolini S, & Hewstone M (2016) Changing people's views of outgroups through individual-to-group generalisation: meta-analytic reviews and theoretical considerations. *European Review of Social Psychology* 27(1):63-115.
15. Smith A, Anderson M (2017) Social Media Use in 2018. (Pew Research Center).
16. Fiske ST, Cuddy AJC, & Glick P (2007) Universal dimensions of social cognition: warmth and competence. *Trends in Cognitive Sciences* 11(2):77-83.
